# Taxonomy and evolution of bacteriochlorophyll *a*-containing members of the OM60/NOR5 clade of marine gammaproteobacteria: description of *Luminiphilus syltensis* gen. nov., sp. nov., reclassification of *Haliea rubra* as *Pseudohaliea rubra gen. nov.,* comb. nov., and emendation of *Chromatocurvus halotolerans*

**DOI:** 10.1186/1471-2180-13-118

**Published:** 2013-05-24

**Authors:** Stefan Spring, Thomas Riedel, Cathrin Spröer, Shi Yan, Jens Harder, Bernhard M Fuchs

**Affiliations:** 1 Leibniz Institute DSMZ, German Collection of Microorganisms and Cell Cultures, Inhoffenstr. 7B, Braunschweig 38124, Germany; 2 Helmholtz-Centre for Infection Research (HZI), Research Group Microbial Communication, Inhoffenstr. 7, Braunschweig 38124, Germany; 3 Department of Molecular Ecology, Max Planck Institute for Marine Microbiology, Celsiusstr. 1, Bremen 28359, Germany; 4 Department of Microbiology, Max Planck Institute for Marine Microbiology, Celsiusstr. 1, Bremen 28359, Germany; 5 Present address: Observatoire Océanologique de Banyuls, Université P. et M. Curie, UMR-CNRS 7621, Laboratoire Arago, Banyuls-sur-Mer 66650, France; 6 Present address: Group of Computational Genetics, CAS-MPG Partner Institute for Computational Biology, SIBS, CAS, Shanghai 200433, China

**Keywords:** Phylogeny, Aerobic anoxygenic photoheterotroph, Proteorhodopsin, Mixotroph, Picoplankton, Coastal marine environment

## Abstract

**Background:**

Aerobic gammaproteobacteria affiliated to the OM60/NOR5 clade are widespread in saline environments and of ecological importance in several marine ecosystems, especially the euphotic zone of coastal areas. Within this group a close relationship between aerobic anoxygenic photoheterotrophs and non-phototrophic members has been found.

**Results:**

Several strains of aerobic red-pigmented bacteria affiliated to the OM60/NOR5 clade were obtained from tidal flat sediment samples at the island of Sylt (North Sea, Germany). Two of the novel isolates, Rap1red and Ivo14^T^, were chosen for an analysis in detail. Strain Rap1red shared a 16S rRNA sequence identity of 99% with the type strain of *Congregibacter litoralis* and was genome-sequenced to reveal the extent of genetic microheterogeneity among closely related strains within this clade. In addition, a draft genome sequence was obtained from the isolate Ivo14^T^, which belongs to the environmental important NOR5-1 lineage that contains so far no cultured representative with a comprehensive description. Strain Ivo14^T^ was characterized using a polyphasic approach and compared with other red-pigmented members of the OM60/NOR5 clade, including *Congregibacter litoralis* DSM 17192^T^, *Haliea rubra* DSM 19751^T^ and *Chromatocurvus halotolerans* DSM 23344^T^. All analyzed strains contained bacteriochlorophyll *a* and spirilloxanthin as photosynthetic pigments. Besides a detailed phenotypic characterization including physiological and chemotaxonomic traits, sequence information based on protein-coding genes and a comparison of draft genome data sets were used to identify possible features characteristic for distinct taxa within this clade.

**Conclusions:**

Comparative sequence analyses of the *pufLM* genes of genome-sequenced representatives of the OM60/NOR5 clade indicated that the photosynthetic apparatus of these species was derived from a common ancestor and not acquired by multiple horizontal gene transfer from phylogenetically distant species. An affiliation of the characterized bacteriochlorophyll *a*-containing strains to different genera was indicated by significant phenotypic differences and *pufLM* nucleotide sequence identity values below 82%. The revealed high genotypic and phenotypic diversity of closely related strains within this phylogenetic group reflects a rapid evolution and frequent niche separation in the OM60/NOR5 clade, which is possibly driven by the necessities of an adaptation to oligotrophic marine habitats.

## Background

Aerobic anoxygenic photoheterotrophic bacteria use light as additional energy source for mixotrophic growth and play a significant role in the microbial ecology of marine environments [1,2]. Members of this physiological group belonging to the *Alphaproteobacteria* have been intensively studied (for review see *e.g.*[3,4]), but so far little is known on the phenotypic diversity of representatives belonging to the *Gammaproteobacteria*. The existence of aerobic anoxygenic photoheterotrophic gammaproteobacteria in marine environments was first postulated in a study by Béjà et al. [5], who could identify photosynthesis genes in partial genome sequences of gammaproteobacteria retrieved from seawater off the coast of California (USA). A few years later the two marine isolates HTCC2080 and KT71^T^ were independently identified as aerobic anoxygenic photoheterotrophic gammaproteobacteria by proteomic analyses [6] and genome sequencing [7], respectively. Strain KT71^T^ was subsequently characterized in detail and described as *Congregibacter litoralis* (*C. litoralis*) by Spring et al. [8], thereby representing the first photoheterotrophic bacterium of this group with a validly published name. Phylogenetically, *C. litoralis* is affiliated to a large coherent cluster of 16S rRNA gene sequences, which were mainly retrieved by cultivation-independent methods from marine habitats around the world. This sequence cluster was recognized as a distinct lineage within the class *Gammaproteobacteria* and designated as OM60 [9,10] or NOR5 clade [11]. Metabolic active bacteria representing this clade could be detected in numerous environmental samples by using fluorescence *in situ* hybridization experiments [12,13]. Based on these findings it is assumed that the OM60/NOR5 clade of *Gammaproteobacteria* is of significant ecological importance due to its widespread occurrence in the euphotic zone of saline ecosystems and high abundance especially in coastal waters [6,13,14]. A phylogenetic lineage closely related to the OM60/NOR5 cluster was originally defined by a 16S rRNA gene sequence retrieved from deep sea sediment and designated BD1-7 [13]. In recent years reports about the isolation of additional strains belonging to the OM60/NOR5 group have accumulated. Some of these strains were described as mixotrophs containing photosynthetic pigments [6,15] or proteorhodopsin (PR) [16]. In contrast, no photosynthetic pigments were reported in members of the genus *Haliea*[17-19] or *Halioglobus*[20]. The recently described non-pigmented species *Dasania marina*[21] and *“Oceanicoccus sagamiensis”*[22] are most likely affiliated to the BD1-7 lineage, whereas representatives of the more distantly related genera *Spongiibacter*[23] and *Zhongshania*[24] form a third phylogenetic branch. In this study, a comprehensive phenotypic and genotypic characterization of the novel isolate Ivo14^T^ was performed that allowed a detailed comparison to other bacteriochlorophyll (BChl) *a*-containing members of the OM60/NOR5 clade, so that a profound knowledge of the metabolic plasticity and taxonomic relationships encountered in this ecologically important group of marine gammaproteobacteria could be obtained.

## Results and discussion

### Isolation and identification of mixotrophic representatives of the OM60/NOR5 clade

An isolation strategy originally designed for the retrieval of strains belonging to the genus *Rhodopirellula* within the *Planctomycetales* resulted in the isolation of numerous representatives of the OM60/NOR5 clade of marine gammaproteobacteria [13,25]. The isolation strategy included the use of antibiotics and a screening of red-pigmented strains, so that all retrieved OM60/NOR5 isolates were pigmented. Strains belonging to this phylogenetic group represented about 10% of all red-pigmented colonies and could be affiliated either to the NOR5-3 or NOR5-1 lineage within this clade based on analyses of their 16S rRNA gene sequences [13]. Strains belonging to the OM60/NOR5 clade were further examined for the presence of *pufL* and *pufM* genes encoding proteins of the photosynthetic reaction center. From 18 out of 22 isolated strains fragments of *pufLM* genes could be amplified by PCR using specific primers. Probably, the strategy of Winkelmann and Harder [25] was such an effective method for the isolation of mixotrophic members of the OM60/NOR5 clade, because it selected for pigmented and slowly growing bacteria adapted to oligotrophic habitats. Two of the isolated strains, Rap1red (= NOR5-3) and Ivo14^T^ (= NOR5-1B^T^), representing two different lineages of the OM60/NOR5 clade were selected for a further analysis using genome sequencing. Strain Ivo14^T^ representing the highly diverse and environmentally important NOR5-1 lineage was chosen for an additional detailed phenotypic characterization.

**Figure 1:**
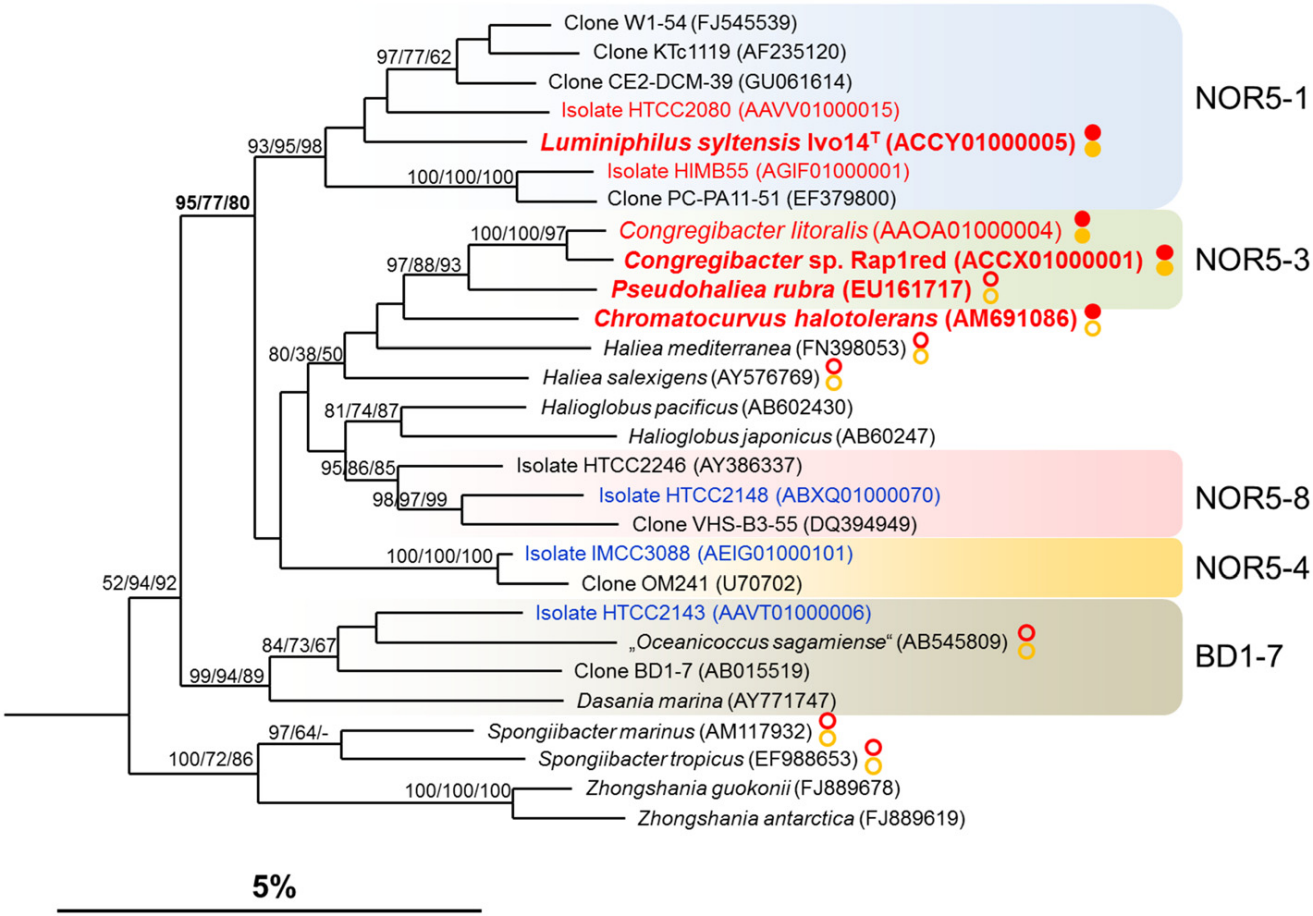
**Phylogenetic tree based on almost complete 16S rRNA gene sequences showing the position of BChl *a*-containing strains within the OM60/NOR5 clade.** The dendrogram was reconstructed with a neighbor-joining distance matrix program as implemented in the ARB package using phylogenetic distances calculated with the algorithm of Jukes and Cantor. No filter or weighting masks were used to constrain the used positions of the alignment. In addition, trees were reconstructed using the PHYLIP maximum parsimony program of ARB and the RAxML maximum likelihood program. Bootstrap values (as percentages of 1000 resamplings) are shown in front of each node, if at least with one reconstruction method a value of 80% or above was obtained. From left to right the numbers indicate results of the neighbor-joining, maximum parsimony and maximum likelihood analyses. A hyphen indicates that the branch was not obtained with the respective reconstruction method. Nucleotide sequence accession numbers are given in parentheses. The affiliation of strains to subclades of the OM60/NOR5 group is based on [13]. The sequence of *Alcanivorax borkumensis* [GenBank:Y12579] was used as outgroup (not shown). Designations given in red color indicate that the respective strains produce BChl *a* and/or encode genes for a photosynthetic apparatus; names in blue indicate the presence of proteorhodopsin encoding genes. Strains that were tested with specific PCR primers for the presence of *pufLM* and *soxB* genes are labeled with red and yellow circles, respectively. Closed circles indicate a positive PCR reaction and open circles a negative reaction. The bar represents an estimated sequence divergence of 5%.

Noteworthy, *Haliea rubra* (*H. rubra)*, which is closely related to *C. litoralis* was also reported to form red-pigmented colonies on Marine Agar 2216 [18], but in the original species description the formation of photosynthetic pigments was not reported. To exclude the possibility that a phototrophic phenotype has escaped attention in described strains of the genus *Haliea*, type strains belonging to this genus were cultured in SYPHC medium, which allowed expression of pigments in all photoheterotrophic strains belonging to the OM60/NOR5 clade tested so far. In fact, photosynthetic pigments could be extracted from cells of *H. rubra*, which showed a pinkish to red pigmentation upon growth in SYPHC medium, whereas no pigments could be extracted from the cream-colored cells of *H. mediterranea* and *H. salexigens*. In order to determine if additional described strains belonging to this clade have unrecognized phototrophic capabilities, extracted DNAs of species that show no visible pigmentation under conditions of laboratory cultivation were used for a PCR screening with specific primers to detect *pufLM* genes. BChl *a*-containing species belonging to the OM60/NOR5 clade were used as positive control. In addition, primers for the detection of *soxB* (representative for a periplasmic enzyme complex oxidizing thiosulfate) and *pop* (gene encoding the opsin subunit of proteorhodopsin) were used to identify alternative potential mixotrophic pathways in described chemoheterotrophic species of the OM60/NOR5 clade and neighboring phylogenetic groups. Results obtained with the *pufLM* and *soxB* primers are depicted in the phylogenetic tree shown in Figure 1. It turned out that the genomic DNA of all species described as non-pigmented (*H. salexigens, H. mediterranea, “Oceanicoccus sagamiensis”, Dasania marina, Spongiibacter tropicus* and *Spongiibacter marinus*) was negative in the amplification of *pufLM* genes, whereas a PCR product of the correct size was obtained from all strains supposed to encode genes for a photosynthetic apparatus, except *H. rubra*. It should be noted that application of the published primers pufLF1 und pufMR1 [5] failed to amplify *pufLM* genes from strain Rap1red, so that we designed the primers pufLF2 und pufMR2, which have a slightly modified sequence optimized for members of the OM60/NOR5 clade. Application of the latter primer set allowed the amplification of the *pufLM* genes of Rap1red and all other available photoheterotrophic members of the OM60/NOR5 clade, but not from *H. rubra* and species described as non-pigmented. However, the *pufLM* nucleotide sequence of *H. rubra* could be finally obtained by the determination of a draft genome sequence (unpublished data). It turned out that at least two mismatches at the binding site of the forward primer prevented a successful amplification of the *pufL* and *pufM* genes from this species.

It was not possible to amplify genes encoding proteorhodopsin or the sulfate thiol esterase SoxB from the non-phototrophic species shown in Figure 1. For the PCR screening with the proteorhodopsin primer set PR1-3 [26] we used genomic DNA from *Dokdonia* sp. PRO95 [27] as well as total DNA isolated from the North Sea as positive control. However, a proteorhodopsin-positive control strain belonging to this phylogenetic group was not available and the *pop* gene sequence of strain IMCC3088 revealed some mismatches to the used proteorhodopsin oligonucleotide primers. Thus, either the tested strains do not encode *pop* genes, or the genes are such different at the primer binding sites that no PCR amplification was possible.

### Phenotypic characterization

#### Morphology of cells and colonies

Size and shape of cells of the newly isolated strain Ivo14^T^ were determined upon growth in SYPHC medium, which was optimal for cultivation of this strain and the related species *C. litoralis*, *H. rubra* and *Chromatocurvus halotolerans*. Cells of Ivo14^T^ were non motile and appeared coccoid or as short straight-to-bent rods. Occurrence of pleomorphic cells was observed in all four BChl *a*-containing strains and depended to some extent on the composition of the growth medium, which makes it important to use the same medium for comparison of size and shape. Especially, growth on the nutrient-rich medium Marine Broth 2216 led in cultures of *H. rubra, C. litoralis* and *Chromatocurvus halotolerans* to cells with irregular shapes, swelling of cells and accumulation of highly refractile storage compounds, whereas these effects were less pronounced in cultures of Ivo14^T^. The storage compound cyanophycin, which is a characteristic of *C. litoralis* was not detected in cells of Ivo14^T^ or *Chromatocurvus halotolerans*, which both accumulate polyhydroxyalkanoates in addition to polyphosphates. The intracellular carbon storage compound of *H. rubra* could be distinguished from cyanophycin or polyhydroxyalkanoates by a positive reaction of the acidified cell extract with the anthrone reagent, which detects carbohydrates. This indicates most likely the presence of glycogen as reserve polymer, because a coherent cluster of glycogen synthesis genes was found in the draft genome sequence of *H. rubra* DSM 19751^T^ (unpublished data).

Under conditions of carbon starvation, cells of *C. litoralis* had a strong tendency to aggregate and to form flocs in liquid medium. Floc formation in this strain is promoted probably by the production and excretion of pili, which can be recognized as meshwork between cells in transmission electron micrographs of cell aggregates (Lünsdorf H., *personal communication*). A similar phenomenon was reported previously for the oligotrophic marine alphaproteobacterium *Candidatus* Pelagibacter ubique [28]. The formation of flocs was also regularly observed in *H. rubra* under conditions of nutrient deprivation and occasionally in *Chromatocurvus halotolerans,* but totally absent in Ivo14^T^.

Colonies of Ivo14^T^ appeared on Marine Agar 2216 after an incubation time of approx. 7 days at 28°C and were dark red, round, concave, smooth and reached a diameter of 1 mm. In contrast, colonies of *C. litoralis* and *Chromatocurvus halotolerans* reached a diameter of approx. 2 mm and appeared already after 3 days. Growth of *H. rubra* on Marine Agar 2216 was strongly inhibited compared to SYPHC agar, so that pin point colonies were only visible after an incubation period of 10 to 14 days. A diffusible brownish pigment produced by strain *Chromatocurvus halotolerans* DSM 23344^T^ was not observed in the strains Ivo14^T^, *H. rubra* DSM 19751^T^ and *C. litoralis* DSM 17192^T^.

#### Photosynthetic apparatus and cytochrome composition

**Figure 2:**
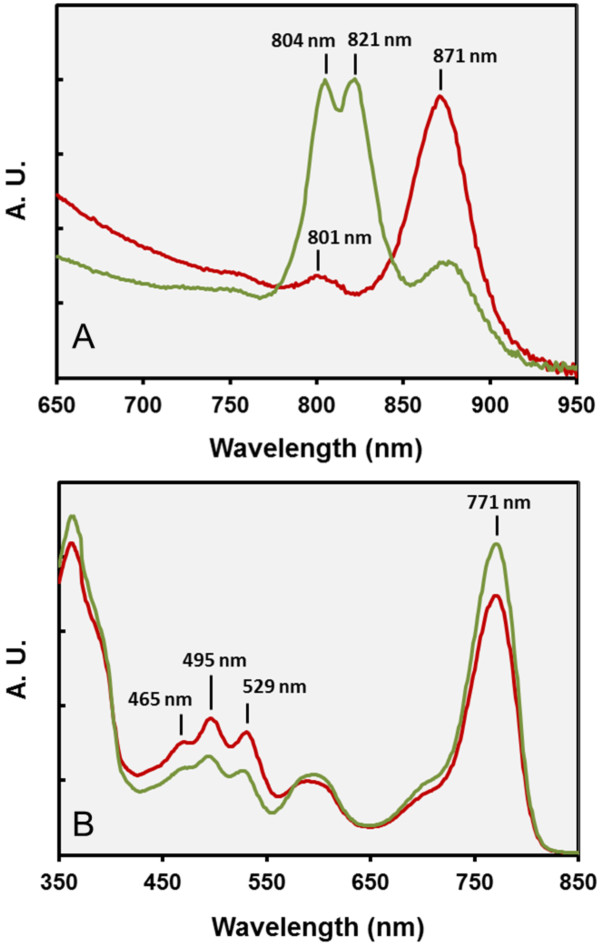
**Spectral characteristics of the photosynthetic apparatus in *Luminiphilus syltensis *Ivo14**^**T **^**and *Pseudohaliea *(= *Haliea*) *rubra *DSM 19751**^**T**^**.** Cells of *Luminiphilus syltensis* Ivo14^T^ (red line) were grown in SYMHC medium in the dark under air atmosphere, while *Pseudohaliea rubra* DSM 19751^T^ (green line) was cultured in SYPHC medium in the light. The position of distinct peaks of the spectra is indicated. A.U., arbitrary units of absorbance. **A.** Whole-cells absorption spectra. **B.** Spectra of acetone/methanol extracts showing the characteristic peaks of BChl *a* and spirilloxanthin.

*In vivo* absorption spectra of pigmented cells of strain Ivo14^T^ revealed near-infrared peaks at 801 and 871 nm, indicating presence of a reaction center embedded in a light-harvesting complex 1 (LH1). No indication of a peripheral LH2 complex was detected in whole-cells absorption spectra (Figure 2A). The near-infrared band of the BChl *a* incorporated in the LH1 complex of Ivo14^T^ was significantly blue–shifted compared to the related species *Chromatocurvus halotolerans* and *C. litoralis*, which displayed peaks at 877 and 876 nm in the respective spectra. Interestingly, the whole-cells spectrum of *H. rubra* showed a clearly distinct profile with major peaks at 804 and 821 nm and only a small peak at 871 nm (Figure 2A). The observed spectrum indicates the presence of a peripheral LH3 complex accompanied by a small amount of the supposed LH1 complex. Light-harvesting complexes of the LH3 type were first described in the purple non-sulfur bacterium *Rhodoblastus acidophilus* incubated under low-light and/or low temperature conditions [29,30]. To the best of our knowledge this is the first report of a LH3 complex in an obligately aerobic anoxygenic phototrophic bacterium. In contrast to *Rhodoblastus acidophilus* the LH3 complex in *H. rubra* was apparently expressed constitutively, because its formation was independent of variations in illumination (light/darkness) or oxygen concentration (6 - 21% (v/v) O_2_ in the headspace gas atmosphere).

UV/visible spectroscopy of acetone/methanol extracts of pigmented Ivo14^T^ cells resulted in peaks that are typical for BChl *a* (363, 600 and 771 nm) and spirilloxanthin (465, 495 and 529 nm). Additional pigments were not observed in this strain. Similar results were obtained for *Chromatocurvus halotolerans*[31] and *H. rubra* DSM 19751^T^ (Figure 2B). Thus, the pigment composition of the photosynthetic apparatus in all obligately aerobic gammaproteobacteria studied so far seems to be identical (Table 1). Maximal levels of pigment expression in Ivo14^T^ were obtained upon incubation in SYMHC medium under air atmosphere. Abundance of the LH1 complex in living cells, estimated by determination of A_870 nm_/A_660 nm_ ratios, reached maximal values of 0.80 to 0.83. This expression level of the LH1 complex corresponded to a measured BChl *a* concentration of around 1.2 nmol/mg cellular dry weight. The obtained results are comparable to values reported for *Chromatocurvus halotolerans*[31], but significantly lower than found in *C. litoralis* which can produce up to 3.5 nmol BChl *a*/mg dry weight under optimal conditions for photoheterotrophic growth [8]. The highest concentration of photosynthetic pigments was however found in *H. rubra*, which could produce up to 4.4 nmol BChl *a*/mg dry weight.

**Table 1:** **Distinguishing features of characterized BChl *a*-containing members of the OM60/NOR5 clade**

**Characteristic**	**1**	**2**	**3**	**4**
**Morphology**				
Size (in SYPHC medium) [μm]	1.2 – 2.2 × 0.6	1.2 – 1.8 × 0.7	1.2 – 1.5 × 0.6	1.2 -1.6 × 0.6
Shape (in SYPHC medium)	straight-to-bent rods, coccoid	straight-to-bent rods, coccoid	straight rods, coccoid	straight rods, coccoid
Storage compounds	PolyP, PHA	PolyP, PHA	PolyP, CP	PolyP, GLY
Motility	-	+	+	-
Cell aggregation	-	w	+	+
**Pigmentation**				
BChl *a* absorption [nm] (*in vivo*)	801, 871	802, 877	802, 876	804, 821, 871
BChl *a* production [nmol/mg dw]	1.2	1.1*	3.5	4.4
Carotenoid absorption [nm] (in acetone/methanol)	465, 495, 529	467, 496, 531	465, 495, 529	470, 496, 530
Diffusible brown compound	-	+	-	-
**Chemotaxonomy**				
Fatty acid 16:1 ω6c	-	-	+	-
Main hydroxy fatty acids (>1% of total fatty acids)	10:0 3OH, 12:0 3OH	11:0 3OH, 12:0 3OH, 12:1 3OH	10:0 3OH	12:1 3OH, 12:0 2OH
Lipoquinones	Q8 (tr. Q7)	Q8	Q8	Q8*
Polar lipids	PG, PE, PN	PG, PE, PL, PN	PG, PE, PL	PG, PE, PL
DNA G + C content [mol%]	57	63*	58	66
**Physiology**				
NaCl range (optimum) [% w/v]	1 – 9 (3)	0 – 18 (4)*	1 – 7 (2)	0.7 – 4.2 (3.5)*
Temp. range (optimum) [°C]	12 – 32 (28)	7 – 40 (37)*	9 – 33 (28)	15 – 44 (30)*
*Antibiotic sensitivity*				
Imipenem (10 μg)	+	-*	+	-
Polymyxin B (300 U)	+	+*	+	-
*Required supplements*				
L-histidine	+	-	-	-
Biotin	+	+*	+	+
Thiamin	+	+*	+	+
Vitamin B12	+	+*	+	+
*Enzyme activities*				
Catalase	+	+	w	+
Oxidase	+	+ [-*]	+	+
Aesculinase	-	-	-	+
Tweenase 20/80	+/w	+/w	+/w	+/+
Urease	-	-	+	-
*Utilization of*				
Sucrose	-	-	+	-
Glycerol	w	-	w	w [-*]
Butanol	+	-	w	+
Propionate	+	+ [-*]	w	+ [-*]
Butyrate	+	+ [-*]	w	+
DL-lactate	+	-	-	+ [-*]
2-oxoglutarate	+	-	+	+
L-serine	-	-	+	+ [-*]
L-proline	-	+	+	-
L-isoleucine	-	+	-	+
L-arginine	-	-	+	-
L-phenylalanine	+	-	-	-
L-glutamate	-	+	+	+ [-*]
L-glutathione	-	+	+	+

All strains were positive in the utilization of acetate, L-alanine, fumarate, DL-3-hydroxybutyrate, DL-malate, oxaloacetate, pyruvate, succinate, and L-threonine. The following compounds were not utilized by all tested strains: citrate, ethanol, formate, D-fructose, D-glucose, glycolate, and methanol. Degradation of starch and gelatin, reduction of nitrate to nitrite and stimulation of growth by thiosulfate were negative in all strains, as well as diagnostic tests for the enzymes tryptophanase and arginine dihydrolase. Data marked with an asterisk were taken from the literature [18,31]. Published data that disagree with our results are shown in brackets. Abbreviations: *PolyP* polyphosphate, *PHA* polyhydroxyalkanoate, *CP* cyanophycin, *GLY* glycogen, *PG* phosphatidylglycerol, *PE* phosphatidylethanolamine, *PL* unidentified phospholipid, *PN* unidentified aminophospholipid, *w* weakly positive reaction. Strains: 1, *Luminiphilus syltensis* Ivo14^T^; 2, *Chromatocurvus halotolerans* DSM 23344^T^; 3, *Congregibacter litoralis* DSM 17192^T^; 4, *Pseudohaliea* (= *Haliea*) *rubra* DSM 19751^T^.

The dominant cytochrome types in pigmented cells of the strains Ivo14^T^, *Chromatocurvus halotolerans* DSM 23344^T^ and *H. rubra* DSM 19751^T^ grown under fully aerobic conditions were determined by redox difference spectroscopy of extracts from whole cells solubilized with the detergent N,N-dimethyldodecylamine-N-oxide (LDAO). In dithionite-reduced *minus* ferricyanide-oxidized redox difference spectra a Soret peak at 421-422 nm and an alpha peak at 553-554 nm indicates that *c*-type cytochromes were dominating. Additional *b*-type cytochromes could be identified by a shoulder of the Soret band around 434 nm in spectra of cell-free extracts of strain Ivo14^T^ and *Chromatocurvus halotolerans* DSM 23344^T^, whereas a shoulder around 445 nm suggests the presence of cytochromes containing heme *a* in Ivo14^T^ and *H. rubra* DSM 19751^T^. A further analysis of the cytochrome composition in these strains is given in [32].

#### Growth characteristics

Growth of strain Ivo14^T^ was observed in the range of pH 7.0 to 9.0 and 12 to 32°C, with an optimum at pH 8.0 and 28°C. The NaCl concentration suitable for growth was 1 - 9% (w/v), the optimum at 3% (w/v). These values were quite similar to that of *C. litoralis* and *H. rubra*, but clearly distinct to *Chromatocurvus halotolerans* (Table 1), which has a higher temperature optimum and is more halotolerant probably due to the adaptation to growth conditions within the microbial mats in a hypersaline spring [31]. Under optimal growth conditions in SYPHC medium the generation time of strain Ivo14^T^ was 13 h and thus quite long compared to the related type strains of *Chromatocurvus halotolerans*, *C. litoralis* and *H. rubra*, which have mean doubling times of 8.7, 4.5 and 3.4 h, respectively. As a peculiarity the requirements of Ivo14^T^ for growth in defined medium were more complex than that of *C. litoralis*, *H. rubra* or *Chromatocurvus halotolerans*. In respect to mineral composition Ivo14^T^ required in addition to sodium chloride, magnesium and calcium ions, whereas *C. litoralis* required besides NaCl only either Mg^2+^ or Ca^2+^. In addition, there seems to be a requirement for certain amino acids. In defined media L-histidine was found to be an essential nutrient for growth of Ivo14^T^. No growth was detected below 40 μmol/l L-histidine in the medium. The growth-stimulating effect was not concentration dependent within the tested range of up to 500 μmol/l. It was also found that L-histidine could be replaced with either L-threonine or L-aspartate, which have completely different pathways of biosynthesis. Interestingly, all three amino acids are common substrates for enzymatic phosphorylation reactions. Consequently, this rather indicates a defect in the global regulation of amino acid synthesis, *e.g.* the stringent response [33,34], than an auxotrophy for certain amino acids. In subsequent experiments a combination of L-histidine and L-cysteine, each in a concentration of 250 μM, was shown to be optimal for growth and expression of photosynthetic pigments in strain Ivo14^T^. L-histidine stimulated also the growth of *H. rubra* in defined media by shortening the observed lag-phase, but it was not an essential compound for growth.

There was no difference in the requirement of vitamins among the four related BChl *a*-containing strains, which all needed biotin, thiamine and B-12. However, some variation in the sensitivity to antibiotics was found. In contrast to *C. litoralis*, strain Ivo14^T^ was resistant to cefalotin, but sensitive to bacitracin and doxycycline. *H. rubra* and *Chromatocurvus halotolerans* could be distinguished from the former two strains by their resistance to imipenem. *H. rubra* was clearly distinct to all strains, because it was only sensitive to chloramphenicol, bacitracin and gentamicin in the applied disk diffusion test encompassing a total of 13 different antibiotics.

#### Substrate utilization pattern and enzyme activities

The utilization of carbon sources and enzyme activities were determined for the novel strain Ivo14^T^ and type strains of the related pigmented species *Chromatocurvus halotolerans* and *H. rubra*. The three strains of BChl *a*-containing aerobic gammaproteobacteria analyzed in this study and *C. litoralis* were markedly different in their substrate utilization patterns, thus enabling their differentiation (see Table 1 and the species descriptions below). Distinguishing characteristics of Ivo14^T^ were the utilization of L-phenylalanine as sole carbon source, whereas L-glutamate and glutathione could not be used. On the other hand, *Chromatocurvus halotolerans* DSM 23344^T^ was unique in the inability to use 2-oxoglutarate and butanol, whereas *H. rubra* DSM 19751^T^ was the only strain expressing the enzyme aesculinase (*β*-glucosidase). The absence of cytochrome *c* oxidase activity in *Chromatocurvus halotolerans*, which was previously postulated as a distinctive trait [31], however could not be confirmed. Based on the comparison of substrate utilization patterns it appears that *C. litoralis* is the metabolic most versatile species being able to utilize a variety of sugars, carboxylic acids and alcohols, probably reflecting frequent changes of the encountered environmental conditions. All four strains were not able to grow under anaerobic or autotrophic conditions in the light, thus confirming their definition as aerobic anoxygenic photoheterotrophic gammaproteobacteria.

It has to be noted that the substrate utilization pattern obtained for *H. rubra* DSM 19751^T^ was significantly different from the one reported previously [18]. The substrates citrate, glucose and lactose could not be utilized (although reported as positive), whereas the substrates acetate, alanine, glutamate, glycerol, lactate, propionate, pyruvate, serine and succinate could be utilized (although reported as negative). In our hands the BIOLOG assay used by Urios et al. [18] for the physiological characterization of *H. rubra* was not satisfactory for photoheterotrophic members of the OM60/NOR5 clade, because neither *H. rubra* DSM 19751^T^ nor *C. litoralis* DSM 17192^T^ or *Chromatocurvus halotolerans* DSM 23344^T^ showed a clear response in BIOLOG plates, at least after an incubation period of 1 - 2 weeks. Thus, it is possible that the deviant results reported elsewhere [18] were caused by using an inappropriate analysis method.

### Chemotaxonomy

The DNA G + C contents of the strains Ivo14^T^ and Rap1red were deduced from the draft genome sequences as 56.7 and 56.3 mol%, respectively. Both values are close to the determined DNA G + C content of *C. litoralis* (57.7 mol% [8]), but significantly lower than in *Chromatocurvus halotolerans* (63 mol% [31]) and *H. rubra* (66.1 mol% determined by genome sequence analysis (this study)). All three strains analyzed in this study possess ubiquinone 8 (Q8) as predominating respiratory lipoquinone, which is typical for obligately aerobic gammaproteobacteria. However, some differences became apparent in the polar lipid pattern. The composition in *C. litoralis* was dominated by phosphatidylglycerol, phosphatidylethanolamine and an unidentified phospholipid [8]. The same pattern was found in *H. rubra* DSM 19751^T^, whereas in Ivo14^T^ an unidentified aminophospholipid instead of the phospholipid was detected. The pattern of *Chromatocurvus halotolerans* DSM 23344^T^ was characterized by an aminophospholipid and an unidentified phospolipid in addition to the dominating polar lipids phosphatidylglycerol and phosphatidylethanolamine (Table 1), so that it could be distinguished from the profiles of Ivo14^T^, *H. rubra* and *C. litoralis*. However, the profile of *Chromatocurvus halotolerans* did match the polar lipid patterns of type strains of the chemoheterotrophic species *H. salexigens* and *H. mediterranea* that were obtained in this study and differed slightly from results published elsewhere [17,19]. The whole-cell fatty acid patterns of the strains Ivo14^T^, *Chromatocurvus halotolerans* DSM 23344^T^ and *H. rubra* DSM 19751^T^ were determined upon growth on Marine Agar 2216 plates. The results were compared with the cellular fatty acid profiles of the type strains of *C. litoralis* and two related chemoheterotrophic *Haliea* species (Table 2). The fatty acid pattern of *H. rubra* DSM 19751^T^ could be distinguished from all other type strains by the low content of 17:0, 17:1 and 10:0 3OH fatty acids, whereas *C. litoralis* DSM 17192^T^ was unique in the synthesis of the unusual 16:1 ω6 unsaturated fatty acid, which suggests an affiliation of both type strains to different genera. Further analyses of the cellular fatty acid profiles of the four BChl *a*-containing strains were performed upon cultivation in SYPHC liquid medium with different oxygen concentrations in the head space gas atmosphere (see Additional file 1). In a previous study it was found that in *C. litoralis* the position of the double bond in the unsaturated fatty acids 16:1 and 18:1 depends on the oxygen saturation and was shifted from the ω7 to the ω6 position under conditions of oxygen limitation [8]. It is known that several pathways for the synthesis of unsaturated fatty acids exist in proteobacteria. An oxygen-dependent pathway is based on desaturases that introduce double bonds in membrane-bound fatty acids by oxidation with molecular oxygen. An alternative oxygen-independent pathway introduces double bonds during elongation of the fatty acid chain [35]. Hence, we propose that *C. litoralis* expresses two distinct desaturases for the fatty acids 16:1 ω7 (Δ9 desaturase, encoded by the proposed gene KT71_07544) and 18:1 ω7 (Δ11 desaturase, probably encoded by KT71_03222), whereas the ω6 unsaturated fatty acids are produced by an oxygen-independent pathway. A similar effect could not be detected in the strains Ivo14^T^, *Chromatocurvus halotolerans* DSM 23344^T^ and *H. rubra* DSM 19751^T^ (Additional file 1). While in the analyzed fatty acid patterns of strain Ivo14^T^ neither the abundance of the unsaturated fatty acids 18:1 ω7 nor 16:1 ω7 correlated with the oxygen saturation, in *Chromatocurvus halotolerans* a decrease of the portion of 18:1 ω7 from 36.6% to 25.8% under conditions of oxygen limitation was detected, which indicates involvement of an oxygen-dependent desaturase. However, in this strain the reduced amount of 18:1 ω7 was not compensated by the formation of 18:1 ω6. The absence of genes encoding putative desaturases in the Ivo14^T^ draft genome suggests that this strain relies completely on an oxygen-independent pathway for the *de novo* synthesis of unsaturated fatty acids. Likewise, *H. rubra* either does not use desaturases for the synthesis of unsaturated fatty acids or the oxygen-independent *de novo* synthesis leads to the common 18:1 ω7 and 16:1 ω7 fatty acids. It should be noted that fatty acid desaturases also can have a function in the cellular defense against oxidative stress. In this way harmful reactive oxygen species are inactivated by the directed oxidation of saturated fatty acid chains within the cytoplasmic membrane. Thus, strains like *C. litoralis* DSM 17192^T^ or *Chromatocurvus halotolerans* DSM 23344^T^ may be better adapted to oxidative stress than Ivo14^T^, which would explain that the negative effect of light on pigment production is most pronounced in strain Ivo14^T^[32]. In a recent study it was shown that in *Dinoroseobacter shibae* the repression of pigment synthesis is mainly caused by oxidative stress [36].

**Table 2:** **Cellular fatty acid patterns of the novel isolate Ivo14**^**T **^**and some related members of the OM60/NOR5 clade**

**Fatty acid**	**1**	**2**	**3**	**4**	**5**	**6**
***Saturated fatty acids***						
10:0	―	―	―	―	―	0.9
11:0	0.6	―	1.0	―	0.8	1.6
12:0	5.0	1.0	2.2	1.1	2.3	1.1
13:0	―	0.9	1.0	―	1.2	1.3
14:0	**5.4**	0.7	2.0	1.8	2.3	2.2
15:0	4.2	**7.4**	4.9	1.0	4.5	**6.6**
15:0 ISO	―	―	―	―	0.6	―
16:0	**24.0**	**8.1**	**5.4**	**26.8**	**5.7**	**11.7**
17:0	3.1	**5.2**	3.1	0.7	**5.8**	**7.0**
18:0	―	―	0.6	0.6	―	―
***Unsaturated fatty acids***						
15:1 ω6c	―	1.8	2.0	―	4.0	1.1
15:1 ω8c	―	1.3	―	―	0.8	2.7
16:1 ω6c	―	―	**6.5**	―	―	―
16:1 ω7c	**36.1**	**21.3**	**23.1**	**24.4**	**26.5**	**18.3**
17:1 ω6c	―	**5.6**	2.8	―	2.3	3.6
17:1 ω8c	―	**19.2**	**8.1**	0.7	**15.4**	**15.3**
18:1 ω7c	**9.7**	**18.0**	**29.7**	**30.0**	**19.3**	**19.3**
19:1 cyc ω8c	―	―	―	―	0.7	―
***Hydroxy fatty acids***						
10:0 3OH	4.8	0.9	2.1	―	2.4	0.8
11:0 3OH	0.6	1.2	―	―	2.5	2.0
12:0 2OH	―	―	―	1.0	―	―
12:0 3OH	2.2	1.1	―	―	1.6	1.3
12:1 3OH	―	1.5	―	2.4	―	―
13:0 3OH	0.7	―	―	―	―	―
**Sum in Feature 7**	1.3	0.8	2.8	―	―	―

Biomass was obtained by growth of cells on Marine Agar 2216 under fully aerobic conditions. Values are percentages of total fatty acids. Major fatty acids (>5% of total amount) are given in bold. Fatty acids that were detected only in trace amounts (0.5% or less of the total amount) are not shown. The position of the double bond in unsaturated fatty acids is located by counting from the methyl (Ω) end of the carbon chain; *cis* isomers are indicated by the suffix c; ISO indicates iso-branched fatty acids. Summed feature 7 contained one or more of the following fatty acids that could not be separated by GLC with the MIDI system: 19:1 ω6c, 19:0 cyc and an unknown fatty acid with an equivalent chain length of 18.846. Strains: 1, *Luminiphilus syltensis* Ivo14^T^; 2, *Chromatocurvus halotolerans* DSM 23344^T^; 3, *Congregibacter litoralis* DSM 17192^T^; 4, *Pseudohaliea* (= *Haliea*) *rubra* DSM 19751^T^; 5, *Haliea salexigens* DSM 19537^T^*;* 6, *Haliea mediterranea* DSM 21924^T^.

### Phylogeny and evolution of the photosynthetic apparatus

Based on 16S rRNA gene identity values the newly isolated strain Ivo14^T^ is only distantly related to described type strains of the OM60/NOR5 clade, including *Halioglobus pacificus* S1-27^T^ (94.6%), *H. rubra* CM41_15a^T^ (94.6%), *C. litoralis* KT71^T^ (94.6%), *H. mediterranea* 7SM29^T^ (94.4%) and *Chromatocurvus halotolerans* EG19^T^ (93.7%). On the other hand, strain Rap1red shows a close phylogenetic relationship with *C. litoralis* KT71^T^ (99.0%) and *H. rubra* CM41_15a^T^ (96.8%), comprising together the NOR5-3 line of descent. In reconstructed phylogenetic trees based on almost complete 16S rRNA gene sequences the genus *Haliea* is currently paraphyletic, because *H. rubra* intermixes with representatives of photoheterotrophic species belonging to the genera *Chromatocurvus* and *Congregibacter*, while it is only distantly related to the type species *H. salexigens* (Figure 1). The type strains of *H. rubra* and *C. litoralis* share a 16S rRNA sequence identity value of 97%, which indicates a close phylogenetic relationship. In several reconstructed phylogenetic trees *Chromatocurvus halotolerans* is positioned adjacent to *C. litoralis* and *H. rubra*, but this affiliation is not supported by significant bootstrap values (Figure 1). Therefore, *Chromatocurvus halotolerans* should not be included in the genus *Congregibacter* or NOR5-3 lineage, which is in line with the suggestion made in a previous work [13].

**Figure 3:**
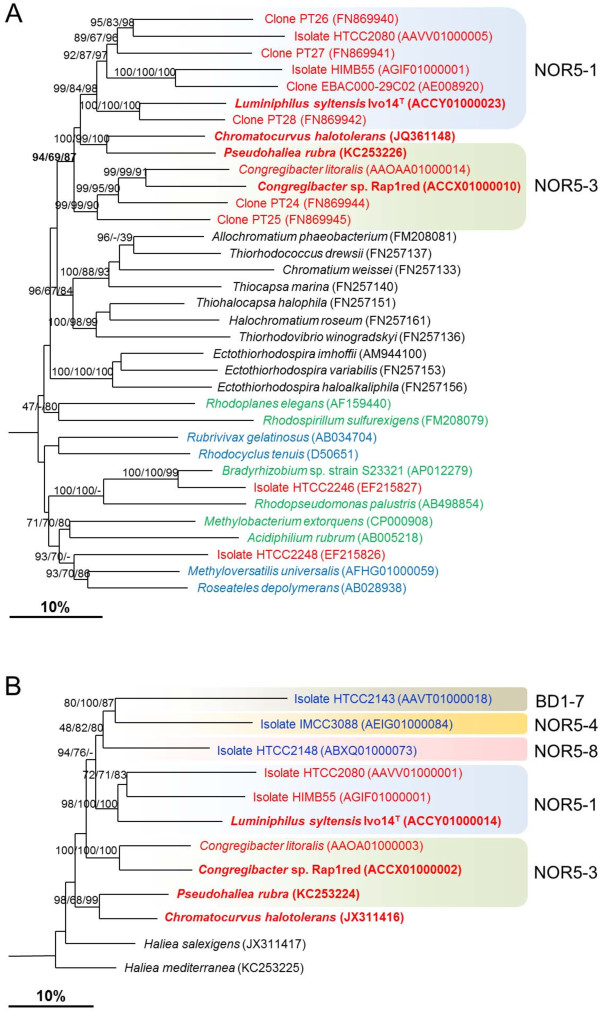
**Reconstruction of phylogenetic relationships among members of the OM60/NOR5 clade based on protein-coding genes.** Phylogenetic trees were reconstructed as outlined in the legend of Figure 1. Size bars represent an estimated sequence divergence of 10%. **A.** Dendrogram based on partial *pufLM* nucleotide sequences. The *pufLM* nucleotide sequence of *Chloroflexus aurantiacus* [GenBank:CP000909] was used as an outgroup (not shown). The red color indicates representatives of the OM60/NOR5 clade, a blue color betaproteobacteria*,* a green color alphaproteobacteria and sequences given in black are affiliated to the order *Chromatiales*. **B.** Dendrogram based on partial *rpoB* nucleotide sequences of members of the OM60/NOR5 clade. Strains known to produce BChl *a* are given in red, names in blue indicate the presence of proteorhodopsin encoding genes. The *rpoB* sequence of *Pseudomomas aeruginosa* PAO1 [GenBank:AE004091] was used as an outgroup.

In Figure 3A a phylogenetic tree based on *pufLM* gene sequences belonging to several distinct groups of *Gammaproteobacteria*, *Betaproteobacteria* and *Alphaproteobacteria* is shown. In this tree sequences of *Chromatocurvus halotolerans* and all genome-sequenced representatives of the OM60/NOR5 clade form a monophyletic group together with several cloned *pufLM* gene sequences retrieved from environmental samples thereby indicating that the photosynthetic reaction center genes within this group were derived from a common ancestor. The topology of *pufLM* gene sequences within the OM60/NOR5 clade is roughly in accordance with the phylogeny derived from 16S rRNA gene data, showing two main branches comprising representatives of the NOR5-1 and NOR5-3 lineages and a third branch represented by *Chromatocurvus halotolerans*. Only the clustering of *H. rubra* with *Chromatocurvus halotolerans* in the *pufLM* based tree represents a discrepancy with the 16S rRNA phylogeny. However, no indications of a horizontal gene transfer of *puf* genes from distant phylogenetic lineages to members of the OM60/NOR5 clade were found, which is in line with results obtained with representatives of the order *Chromatiales*, a group of purple sulfur bacteria belonging to the *Gammaproteobacteria*[37]. This is in contrast to the *Alphaproteobacteria* and *Betaproteobacteria,* in which apparently horizontal gene transfer of *pufL* and *pufM* genes among phototrophic members has occurred (Figure 3A). One possible explanation for this divergence could be the variable genome structure in some members of the *Alphaproteobacteria*, especially the *Roseobacter* clade [38]. However, a subsequent loss of photosynthesis genes or horizontal transfer of photosynthesis genes within the OM60/NOR5 clade is still possible, thereby explaining the close relationship of phototrophic and non-phototrophic species within this group. Nevertheless, our results contradict a previous report postulating a polyphyletic origin of photosynthetic reaction center genes in members of the OM60/NOR5 clade based on results obtained with the strains HTCC2148 and HTCC2246 [6]. In the meanwhile, a draft genome sequence of HTCC2148 has been determined [39], but *pufLM* gene fragments identified by PCR in a previous report [6] were missing. Currently, no genome sequence of strain HTCC2246 is available, but it belongs like HTCC2148 to the NOR5-8 branch within the OM60/NOR5 clade, which does not contain any known phototrophic representatives so far (Figure 1). In addition, we found in our analysis a high similarity of the *pufLM* genes of HTCC2246 with the *Bradyrhizobium* sp. strain S23321 (Figure 3A). *Bradyrhizobium* species are found in the rhizosphere of plants where they form root nodules. Hence, the *pufLM* genes of strain HTCC2246 must have been recently transferred from a nitrogen-fixing, soil bacterium forming root-nodules. However, this would be highly unlikely, because strain HTCC2246 like most other known members of the OM60/NOR5 clade is a marine bacterium, which was isolated from the open sea water and not from soil. Consequently, we speculate that the results reported by Cho et al. [6] may have been caused by a contamination of the analyzed samples with cells or DNA of phototrophic alpha- or betaproteobacteria inhabiting freshwater or soil, but not marine environments.

In terms of evolution, the acquisition of genes for anoxygenic photosynthesis in aerobic gammaproteobacteria could have occurred in members of the NOR5-3 lineage, which still contain genes encoding a peripheral LH2 complex (see Tables 1 and 3) and possess an intracytoplasmic membrane system [8] which is typically found in facultative anaerobic photosynthetic purple bacteria, but is otherwise quite uncommon in aerobic anoxygenic photoheterotrophic bacteria [4]. Possibly, a further adaptation of the photosynthetic apparatus to the conditions of aerobic marine environments in members of the NOR5-1 lineage led to a rapid diversification and speciation process in this subclade, reflected by a high number of microdiverse 16S rRNA gene sequences retrieved from marine surface waters. Probably, the optimization of anoxygenic photophosphorylation under aerobic conditions gave representatives of the NOR5-1 lineage a selective advantage, which enabled them to play a significant role in the euphotic zone of coastal marine environments. An evolving specialization to a distinct type of metabolism could be also reflected in the observed reduction of the genome size among photoheterotrophic members of the OM60/NOR5 clade: The genomes of *C. litoralis* and Rap1red have an estimated size of 4.3 and 4.2 million base pairs (Mb), whereas in the strains HTCC2080, Ivo14^T^ and Himb55, which all belong to the NOR5-1 lineage considerably smaller genome sizes of 3.6, 3.3 and 2.7 Mb, respectively, were found. Previously, it was claimed that reductive genome evolution in the genera *Prochlorococcus* and *Candidatus* Pelagibacter is driven by an adaptation to the oligotrophic growth conditions in open ocean waters [40,41].

**Table 3:** Presence of genes with taxonomic significance in members of the OM60/NOR5 and BD1-7 clades

**Signature genes**	**Putative phenotypic trait**	**NOR5-1**		**NOR5-3**		**NOR5-4**	**BD1-7**
		**1**	**2**	**3**	**4**	**5**	**6**
*pufLMC*	Photosynthetic reaction center	+	+	+	+	-	-
*pucAB*	Light-harvesting complex 2	-	-	+	+	-	-
*ppsR*	Repression of pigment synthesis	+	+	+	+	-	-
*BLUF*	Response to blue light	+	+	+	+	+	-
*pop*	Proteorhodopsin	-	-	-	-	+	+
*soxB*	Thiosulfate oxidation	+	+	+	+	+	-
*ctaCDGE*	*caa*_3_ cytochrome *c* oxidase	+	+	+	+	+	+
*ccoNOQP*	*cbb*_3_ cytochrome *c* oxidase	+	+	+	+	+	+
*cydAB*	Cytochrome *bd*2 quinol oxidase	-	-	-	+	-	-
*flhOPQRBA*	Motility	-	+	+	+	+	+
*pilMNOPQ*	Type IV pili	+	+	+	+	+	+
*cphAB*	Cyanophycin production	-	-	+	+	-	-
*ppk*	Polyphosphate storage	+	+	+	+	+	-
*phaBC*	Polyhydroxyalkanoate production	+	-	-	-	-	-
*desC*	Oxygen-dependent synthesis of monounsaturated fatty acids	-	+	+	+	+	+
*sod*	Superoxide dismutase	+	-	+	+	+	-
*katG*	Catalase/Peroxidase	+	+	+	+	+	-
*ureABC*	Urease	-	-	+	+	-	-
*bglx*	Beta-glucosidase	-	+	-	+	+	+
*paaNBDFGHIJK*	Aromatic ring cleavage	+	+	-	-	-	-

The affiliation of strains to subclades is based on [13]. Strains and accession numbers: 1, *Luminiphilus syltensis* Ivo14^T^ [GenBank:ACCY01000000]; 2, marine gammaproteobacterium HTCC 2080 [GenBank:AAVV01000000]; 3, *Congregibacter litoralis* KT71^T^ [GenBank:AAOA01000000]; 4, *Congregibacter* sp. Rap1red [GenBank:ACCX01000000]; 5, gammaproteobacterium IMCC3088 [GenBank:AEIG01000000]; 6, marine gammaproteobacterium HTCC2143 [GenBank:NZ_AAVT00000000].

On the other hand, the known closely related non-phototrophic species do not represent large coherent clusters of environmental 16S rRNA gene sequences, but rather belong to lineages comprising 16S rRNA gene sequences that are less frequently found in the environment. Possible reasons could be that they remained either dependent on nutrient-rich sites for successful proliferation or are specialized on recalcitrant carbon sources [42] resulting in a more restricted distribution and lower frequency in sea water. Furthermore, it can be concluded that the acquisition of *sox* (thiosulfate oxidation) or *pop* (proteorhodopsin) genes had not the same effect on the diversification and expansion of the respective strains as the acquisition of photosynthesis genes. No growth stimulating effect was detected upon supplementation of media with thiosulfate, so that *sox* genes in these species may have a different function that does not correlate with mixotrophy. The situation for proteorhodopsin is more complicated, because no data about the effect of light on the growth response of PR-harboring strains belonging to the OM60/NOR5 clade (*e.g.* IMCC3088) are currently available. However, it can be assumed that unlike BChl *a*-dependent photophosphorylation that allows an increase of growth yield by the utilization of light [8,32], light-driven proton pumping by membrane-embedded proteorhodopsin does not have this effect, at least in the marine alpha- and gammaproteobacteria studied so far [43,44]. According to current knowledge proteorhodopsin in marine proteobacteria only helps to survive periods of starvation, *i.e.* in the absence of a suitable carbon source or essential nutrients like iron or phosphorous, but does not promote proliferation in cases when the amount of an available carbon source limits growth [28,45]. This could also explain, why the proteorhodopsin-containing alphaproteobacterium *Candidatus* Pelagibacter ubique is dominating in extreme oligotrophic nutrient depleted surface waters in the middle of the oceans [46], whereas aerobic anoxygenic photoheterotrophic gammaproteobacteria prevail in coastal surface waters [14,47,48], where in most cases the amount of the carbon source is the growth limiting factor.

### A taxonomic framework for the OM60/NOR5 clade based on phylogenomic data

#### Delineation of species

An established approach for the delineation of species is the comparison of whole genome data, for example by calculating the overall similarity using high-scoring segment pairs (HSPs). The HSP method is implemented in the Genome-to-Genome Distance Calculator (GGDC), which infers distances from the comparison of a set of HSPs using three distinct formulas. The obtained distances can then be transformed to values analogous to experimentally obtained DNA-DNA similarity values, which still represent a widely accepted gold standard for the delineation of species in bacterial taxonomy [49]. According to the GGDC the estimated DNA-DNA similarity value between the two most closely related strains *C. litoralis* DSM 17192^T^ and Rap1red was only 19.8% (± 8.1%) and thus clearly below 70%, which is the widely accepted threshold value for assigning strains to the same species. The low calculated overall genome similarity is in good agreement with the observed high sequence divergence of protein-coding genes, which exclude an affiliation of both strains to the same species despite the high 16S rRNA gene identity value of 99%. Although, the 16S RNA gene identity value between the type strains of *C. litoralis* and *H. rubra* is only 97%, it is close to the traditionally used threshold value above which the affiliation of strains to the same species should be tested by DNA-DNA similarity experiments [50]. We determined the level of DNA-DNA relatedness between *C. litoralis* and *H. rubra* in a wet lab DNA-DNA reassociation experiment. The obtained result was 21.3% (average of two measurements) and hence as expected below the threshold value of 70%.

#### Delineation of genera

In bacterial taxonomy the definition of genera is more complicated than the classification of species, because universal applicable threshold values still do not exist. The 16S rRNA gene identity values observed among cultured members of the OM60/NOR5 clade range from 91 to 99% with low divergence values between chemoheterotrophic and photoheterotrophic representatives. In some phylogenetic groups, like *Mycoplasmatales* (*e.g.*, [51]) or *Spirochaetales* (*e.g.*, [52]) such values are typically found among members of a single genus, which may be due to the restricted number of suitable phenotypic traits available for classification among the members of these phylogenetic groups. On the other hand, in families that are phenotypically well studied, like *Chromatiaceae* (*e.g.*, [53]) or *Enterobacteriaceae*[54] the delineation of genera is often based on 16S rRNA gene divergence values of around 3% or less. However, the determined significant phenotypic differences among closely related strains within the OM60/NOR5 clade indicate that comparative 16S rRNA sequence analyses alone do not allow a reliable dissection of taxa in this phylogenetic group. In such cases, comparative sequence analyses of housekeeping genes is often used as alternative to 16S rRNA gene analyses to obtain a more reliable discrimination of taxa, because protein-coding genes are less conserved in evolution than the 16S rRNA gene, so that a better resolution of closely related species can be obtained. In addition, a comparison of protein-coding genes avoids the bias of arbitrarily selected phenotypic traits often used for the characterization of species. Previously, sequences of *pufL* and *pufM* genes encoding subunits of the photosynthetic reaction center were successfully used to deduce phylogenetic relationships among phototrophic purple sulfur bacteria (*Chromatiales*) [37]. It was found that a classification to the genus level is possible based on partial nucleotide sequences of *pufL* and *pufM* genes. In that study *pufLM* nucleotide sequence identity values below 86% and 81% correlated with membership to different genera in *Chromatiaceae* and *Ectothiorhodospiraceae*, respectively [37]. Applying the lower threshold value to the OM60/NOR5 clade, it turns out that only the closely related strains *C. litoralis* DSM17192^T^ and Rap1red belong to the same genus, sharing a *pufLM* nucleotide sequence identity value of 82.7%. The *pufLM* genes of the two strains *H. rubra* DSM 19751^T^ [GenBank:KC253226] and *Chromatocurvus halotolerans* DSM 23344^T^ [GenBank:JX311416] have a sequence identity of 80.7%, but an affiliation of both strains to the same genus would be in contradiction to phenotypic and 16S rRNA sequence data. Among all other photoheterotrophic representatives of this clade the *pufLM* sequence identity values are in the range between 69.3 and 76.6% and hence clearly below the genus level. For instance, the identity level of the *pufLM* genes of the two strains Ivo14^T^ and HTCC2080 is only 73.6%, despite a close relationship at the 16S rRNA gene sequence level (96.1%).

The high divergence values of the *pufLM* genes could either indicate a rapid evolution of the photosynthetic apparatus alone or of the total genome. In order to determine representative levels of genome divergence, we have selected the housekeeping gene *rpoB* encoding the RNA polymerase *β-*subunit as an additional phylogenetic marker. It is assumed that the *rpoB* gene is representative for the total genome and thus can be used for the delineation of species and genera [55]. Despite some minor variations depending on the analyzed phylogenetic group, the proposed value for the *rpoB* gene sequence identity level of strains belonging to the same species is above 98% and for species of a single genus above approx. 85% [54,56]. Accordingly, the *rpoB* nucleotide sequence identity between the strains *C. litoralis* DSM 17192^T^ and Rap1red (84.9%) would indicate an affiliation to the same genus, whereas all other values determined among genome sequenced members of the OM60/NOR5 clade were below 80% (72.2-77.8%), which is in good agreement with conclusions deduced from the *pufLM* sequence identity values. Furthermore, partial *rpoB* nucleotide sequences of type strains of the species *H. salexigens* [GenBank:JX311417], *H. mediterranea* [GenBank:KC253225] and *Chromatocurvus halotolerans* [GenBank:JX311416] were determined upon retrieval by PCR amplification, while a complete *rpoB* gene sequence was extracted from the unpublished draft genome of *H. rubra* DSM 19751^T^ [GenBank:KC253224]. A comparison of the determined sequences with the available *rpoB* data set revealed that all identity values were below 85%, except between *H. rubra* and *Chromatocurvus halotolerans*, which share an *rpoB* gene sequence identity value of 86.5%. This value is unusually high compared to an *rpoB* sequence identity value of 80.1% between *H. rubra* and *C. litoralis,* which even share a higher 16S rRNA gene identity of 97.0%. A phylogenetic diagram based on the partial *rpoB* nucleotide sequences (Figure 3B) confirms with high bootstrap support the existence of two main lineages (NOR5-1 and NOR5-3) of BChl *a*-containing strains within the OM60/NOR5 clade. The clustering of *H. rubra* with *Chromatocurvus halotolerans* confirms the results obtained by comparison of the *pufLM* genes, but is in conflict with the 16S rRNA based phylogenetic tree. Probably, the observed highly divergent *pufLM* and *rpoB* nucleotide sequences among closely related members of the OM60/NOR5 clade indicate that the genomes of these bacteria undergo rapid evolution, which may not be reflected in corresponding changes of the highly conserved 16S rRNA gene sequences.

With the exception of *C. litoralis* DSM 17192^T^ and Ivo14^T^ all other genome sequenced isolates belonging to the OM60/NOR5 and BD1-7 clades have not yet been characterized phenotypically in detail. However, distinguishing phenotypic features are still a requirement for the formal description of novel taxa. Therefore, we analyzed the available genome data for the presence of genes with a potential taxonomic significance, *i.e.* encoding traits that could be useful for the description of species and genera. The results of our analyses are shown in Table 3 and it turned out that both strains Rap1red and *C. litoralis* DSM 17192^T^ can be distinguished from other members of the analyzed phylogenetic group based on traits that are not strain or species specific. Among members of the OM60/NOR5 clade genes for urease and cyanophycin synthetase are so far only found in the latter two strains and can therefore be used for the delineation of the genus *Congregibacter* from other BChl *a*-containing taxa.

## Conclusions

In summary, molecular and phenotypic data support the affiliation of the photoheterotrophic strains Ivo14^T^, *Chromatocurvus halotolerans* DSM 23344^T^, *H. rubra* DSM 19751^T^ and *C. litoralis* DSM 17192^T^ to different genera within the OM60/NOR5 clade. In addition, the detection of a photosynthetic apparatus in *H. rubra* suggests its separation from the non-phototrophic genus *Haliea*. A formal description of strain Ivo14^T^ as novel genus and species, the reclassification of *H. rubra* as *Pseudohaliea rubra* and an emendation of the description of *Chromatocurvus halotolerans* follow below.

### Description of *Luminiphilus* gen. nov

*Luminiphilus* (Lu.mi.ni’phi.lus. L. n. *lumen -inis*, light; N.L. masc. adj. *philus* (from Gr. masc. adj. *philos*), friend, loving; N.L. masc. n. *Luminiphilus*, bacterium loving light, referring to the utilization of light for the promotion of growth).

Cells are Gram-negative, non-spore-forming and multiply by binary fission. Mesophilic and moderately halophilic. Strictly aerobic, respiratory and heterotrophic metabolism. In liquid medium large cell aggregates are not observed, even under conditions of carbon starvation. Cyanophycin is not produced as storage material. Tests for oxidase and catalase activity are positive. Cytochromes of the *c*-type are dominating in redox difference spectra. BChl *a* and carotenoids of the spirilloxanthin series are produced in variable amounts depending on the incubation conditions. Does not produce urease, arginine dihydrolase, tryptophanase or aesculinase. Nitrate is not reduced to nitrite. Major cellular fatty acids are C_16:0_, C_16:1_ and C_18:1_. The dominating hydroxy fatty acids are C_10:0_ 3OH and C_12:0_ 3OH. Phosphatidylglycerol, phosphatidylethanolamine and an unidentified aminophospholipid are the major polar lipids. Ubiquinone 8 is the dominating respiratory lipoquinone. Representatives can be found in seawater and the surface layer of littoral marine sediments.

The type species is *Luminiphilus syltensis*.

### Description of *Luminiphilus syltensis* sp. nov

*Luminiphilus syltensis* (sylt.en’sis. N.L. masc. adj. *syltensis*, of or pertaining to the Sylt island, the region of origin).

In addition to traits noted for the genus the following characteristics were determined.

Cells are non-motile straight-to-bent rods which have a tendency to form coccoid or pleomorphic shapes. The dimensions of cells grown in SYPHC medium varies between 1.2 and 2.2 μm in length and 0.6 μm in width. Intracellular storage compounds are polyphosphate and polyhydroxyalkanoates. Colonies appear after about 7 days on plates of Marine Agar 2216 and are round, concave, smooth and dark red. The *in vivo* absorption of BChl *a* in the near-infrared region of the spectrum shows peaks at 801 and 871 nm, indicating the presence of a reaction center and light-harvesting complex 1. Optimal growth conditions are at 28°C, pH 8 and a salinity of approx. 3% (w/v) NaCl. The tolerated salinity for growth ranges from 1 – 9% (w/v) NaCl. The mean generation time under optimal growth conditions is 13 h. Besides NaCl, magnesium and calcium ions are required for growth. The nutrients biotin, thiamin, vitamin B_12_ and L-histidine are essential for growth in mineral medium. L-histidine can be replaced by the amino acids L-threonine or L-aspartate. Sensitive to the antibiotics imipenem, chloramphenicol, gentamicin, neomycin, doxycycline, colistin, polymyxin B and bacitracin; resistant to cephalotin, oxacillin, tetracycline, vancomycin and lincomycin. The polymers alginate, agar, casein, cellulose, DNA, gelatin and starch are not degraded, but Tween 20 is hydrolyzed. The following compounds are used for growth: acetate, L-alanine, butanol, butyrate, dodecanoate, fumarate, glycerol (weak), hexanoate, DL-3-hydroxybutyrate, DL-lactate, DL-malate, octanoate, oleate, oxaloacetate, 2-oxoglutarate, palmitate, L-phenylalanine, propionate, pyruvate, succinate, L-threonine, and valerate. The following compounds were tested, but not utilized: acrylate, 2-aminobenzoate, L-arabinose, L-arginine, L-asparagine, L-aspartate, benzoate, cellobiose, citrate, n-decane, decanoate, meso-erythritol, ethanol, formate, D-fructose, D-galactose, D-glucose, L-glutamate, glutathione, DL-glycine, glycolate, n-hexadecane, L-histidine, myo-inositol, L-isoleucine, D-lactose, L-leucine, L-lysine, D-maltose, D-mannitol, D-melibiose, methanol, L-methionine, n-octane, L-ornithine, 3-phenylpropionic acid, L-proline, propanol, resorcinol, L-rhamnose, L-serine, sucrose, taurine, L-tryptophan, L-valine, and D-xylose. Thiosulfate does not stimulate growth.

The major cellular fatty acids upon culturing on plates of Marine Agar 2216 under fully aerobic conditions are C_16:1_ω7c, C_16:0_, C_18:1_ω7c, and C_14:0_. The DNA G + C content of the type strain is 56.7 mol% (determined from the genome sequence).

The type strain is Ivo14^T^ (= NOR5-1B^T^ = DSM 22749^T^ = JCM 17770^T^). It was isolated from the top oxic layer of a muddy littoral sediment close to the island of Sylt (North Sea, Germany).

### Description of *Pseudohaliea* gen. nov

*Pseudohaliea* (Pseu.do.ha’lie.a. Gr. adj. *pseudês*, false; N.L. fem. n. *Haliea,* a bacterial genus name*;* N.L. fem. n. *Pseudohaliea*, false *Haliea*)

Cells are Gram-negative, non-spore-forming and multiply by binary fission. Mesophilic and moderately halophilic. Strictly aerobic, respiratory and heterotrophic metabolism. Cyanophycin is not produced as storage material. Tests for oxidase and catalase activity are positive. Cytochromes of the *c*-type are dominating in redox difference spectra. BChl *a* and carotenoids of the spirilloxanthin series are produced in variable amounts depending on the incubation conditions. Does not produce urease, arginine dihydrolase or tryptophanase. Nitrate is not reduced to nitrite. Major cellular fatty acids are C_16:0_, C_16:1_ and C_18:1_. The dominating hydroxy fatty acids are C_12:0_ 2OH and C_12:1_ 3OH. Phosphatidylglycerol, phosphatidylethanolamine and an unidentified phospholipid are the major polar lipids. Ubiquinone 8 is the dominating respiratory lipoquinone. Representatives are mainly found in seawater.

The type species is *Pseudohaliea rubra*.

### Description of *Pseudohaliea rubra* comb. nov

*Pseudohaliea rubra* (ru’bra. L. fem. adj. *rubra*, red).

Basonym: *Haliea rubra* Urios et al. 2009

The description of the species is based on the information provided in [18] and this study.

Cells are non-motile straight rods which have the tendency to form coccoid or pleomorphic shapes. The dimensions of cells grown in SYPHC medium varies between 1.2 and 1.6 μm in length and 0.6 μm in width. Intracellular storage compounds are polyphosphate and glycogen. Cells have a tendency to form aggregates in liquid medium. Colonies appear after about 10 to 14 days on plates of Marine Agar 2216 and are round, concave, smooth and dark red. The *in vivo* absorption of BChl *a* in the near-infrared region of the spectrum shows two main peaks at 804 and 821 nm and a minor peak at 871 nm, indicating the presence of a light-harvesting complex 3 along with small amounts of a light-harvesting complex 1. Optimal growth conditions are at 30°C, pH 8 and a salinity of approx. 3.5% (w/v) NaCl. The tolerated salinity for growth ranges from 0.7 – 4.2% (w/v) NaCl. The mean generation time under optimal growth conditions is 3.4 h. The vitamins biotin, thiamin and B_12_ are essential for growth in mineral medium. Sensitive to the antibiotics chloramphenicol, gentamicin and bacitracin; resistant to cephalotin, imipenem, neomycin, colistin, polymyxin B, oxacillin, tetracycline, doxycycline, vancomycin and lincomycin. The polymers agar, gelatin and starch are not degraded, but Tween 20 and Tween 80 are hydrolyzed. The following compounds are used for growth: acetate, L-alanine, butanol, butyrate, fumarate, L-glutamate, glutathione, glycerol (weak), DL-3-hydroxybutyrate, L-isoleucine, DL-lactate, DL-malate, oxaloacetate, 2-oxoglutarate, propionate, pyruvate, L-serine, succinate and L-threonine. The following compounds were tested, but not utilized: L-arabinose, L-arginine, citrate, ethanol, formate, D-fructose, D-galactose, D-glucose, glycolate, D-lactose, D-maltose, D-mannose, methanol, L-phenylalanine, L-proline and sucrose. Thiosulfate does not stimulate growth. Aesculinase is produced.

The major cellular fatty acids upon culturing on plates of Marine Agar 2216 under fully aerobic conditions are C_18:1_ω7c, C_16:0_ and C_16:1_ω7c. The DNA G + C content of the type strain is 66 mol% (determined from the genome sequence).

The type strain is CM41_15a^T^ (=DSM 19751^T^ = CIP 109758^T^ = MOLA 104^T^), which was isolated from surface seawater in the bay of Banyuls-sur-Mer (42 ° 29′ N 3° 08′ E).

### Emended description of the genus *Chromatocurvus* corrig. Csotonyi et al. 2012

The description is based on the data presented in [31] and this study. The corrected name was validly published in [57].

Cells are Gram-negative, non-spore-forming and multiply by binary fission. Mesophilic and moderately halophilic. Strictly aerobic, respiratory and heterotrophic metabolism. Cyanophycin is not produced as storage material. Tests for oxidase and catalase activity are positive. Cytochromes of the *c*-type are dominating in redox difference spectra. BChl *a* and carotenoids of the spirilloxanthin series are produced in variable amounts depending on the incubation conditions. Does not produce urease, arginine dihydrolase, tryptophanase or aesculinase. Nitrate is not reduced to nitrite. Major cellular fatty acids are C_16:0_, C_16:1_ and C_18:1_. The dominating hydroxy fatty acids are C_11:0_ 3OH, C_12:0_ 3OH and C_12:1_ 3OH. Phosphatidylglycerol, phosphatidylethanolamine, an unidentified phospholipid and an unidentified aminophospholipid are the major polar lipids. Ubiquinone 8 represents the sole respiratory lipoquinone. The first isolated representative was obtained from a hypersaline mat of a brine spring in Canada.

The type species is *Chromatocurvus halotolerans*.

### Emended description of *Chromatocurvus halotolerans* corrig. Csotonyi et al. 2012

The characteristics of this species are as described in [31] with the following additions and modifications.

Intracellular storage compounds are polyphosphate and polyhydroxyalkanoates. The mean generation time under optimal growth conditions is 8.7 h. Substrates utilized for growth are acetate, L-alanine, butyrate, fumarate, L-glutamate, glutathione, DL-3-hydroxybutyrate, L-isoleucine, DL- malate, oxaloacetate, L-proline, propionate, pyruvate, succinate and L-threonine. The following compounds were tested, but do not support growth: L-arginine, butanol, citrate, ethanol, formate, D-fructose, D-glucose, glycerol, glycolate, DL-lactate, methanol, 2-oxoglutarate, L-phenylalanine, L-serine and sucrose. Thiosulfate does not stimulate growth.

The major cellular fatty acids upon culturing on plates of Marine Agar under fully aerobic conditions are C_16:1_ω7c, C_17:1_ω8c, C_18:1_ω7c, C_16:0_, C_15:0_, C_17:1_ω6c, and C_17:0_.

## Methods

### Source of sample and isolation procedure

The general isolation procedure has been already described in a previous report [25], which was however focused mainly on the isolation of *Rhodopirellula* strains. In brief, the OM60/NOR5 isolates were obtained as follows: In October 2005 sediment samples were collected from a tidal flat area at Königshafen bay, near the town of List on the German Island of Sylt. The approx. geographic coordinates of the sampling site were 55.04° North and 8.42° East. Most samples were obtained from the top oxic layer of muddy or sandy intertidal sediments. After transportation to the laboratory additional 1:10 and 1:100 dilutions of the original sediment samples were prepared in artificial seawater, then 50 or 200 μl aliquots of each sample were spread on agar plates of Pla-rich medium supplemented with the antibiotics ampicillin and cycloheximide added in a concentration of 2.0 g/l each. The exact composition of Pla-rich medium has already been described elsewhere [25]; essentially it is composed of artificial seawater supplemented with vitamins and trace elements that contains 0.25 g/l each of yeast extract, peptone and glucose as substrates. Colonies displaying a pinkish to red-violet pigmentation appeared after several weeks of incubation at 24°C. Pigmented colonies were further purified by subsequent transfers on Pla-rich agar plates without antibiotics. To determine purity and the phylogenetic affiliation of isolated strains the 16S rRNA genes were PCR-amplified from whole cells and then directly sequenced using an ABI 3130*xl* DNA sequencer (Applied Biosystems; Darmstadt, Germany). A total of 240 red-pigmented colonies were obtained, of which 22 could be affiliated to the OM60/NOR5 clade by phylogenetic analyses of their partial 16S rRNA gene sequences.

### Used strains, media and cultivation conditions

In addition to the newly isolated strains Rap1red and Ivo14^T^ the following reference strains were taken from the DSMZ culture collection and used for comparison: *Chromatocurvus halotolerans* DSM 23344^T^ (= EG19^T^), *Congregibacter litoralis* DSM 17192^T^ (= KT71^T^), *Cronobacter muytjensii* DSM 21870^T^ (= ATCC 51329^T^), *Dasania marina* DSM 21967^T^ (= KOPRI 20902^T^), *Haliea mediterranea* DSM 21924^T^ (= 7SM29^T^), *Haliea rubra* DSM 19751^T^ (= CM41_15a^T^), *Haliea salexigens* DSM 19537^T^ (= 3x/A02/235^T^), “*Oceanicoccus sagamiensis*” DSM 25275^T^ (= PZ-5^T^), *Spongiibacter marinus* DSM 17750^T^ (= HAL40b^T^) and *Spongiibacter tropicus* DSM 19543^T^ (= CL-CB221^T^). *Haliea rubra* CM41_15a^T^ was deposited in the DSMZ by the Laboratoire Arago, Université Pierre et Marie Curie (Banyuls-sur-mer, France) under the conditions of a Material Transfer Agreement. The authenticity of the used strains has been confirmed by the Identification Service of the DSMZ by sequencing of the respective 16S rRNA genes.

For routine cultivation all strains were grown on Marine Broth or Agar 2216. The BChl *a*-containing strains Ivo14^T^, DSM 17192^T^, DSM 19751^T^ and DSM 23344^T^ were also grown in a complex medium that was less nutrient-rich and more suitable for the expression of photosynthetic pigments in these strains. It was designated SYPHC medium and has the following composition (per liter demineralized water): 35.00 g sea salts, 0.10 g NH_4_Cl, 0.05 g KH_2_PO4, 2.50 g HEPES (4-(2-hydroxyethyl)-1-piperazineethanesulfonic acid), 1.00 g yeast extract, 1.10 g sodium pyruvate, 0.04 g L-histidine, 0.04 g L-cysteine-HCl × H_2_O, 1.00 ml Wolfe’s mineral elixir (see DSMZ medium 792) [58], and 1.00 ml vitamins solution (see DSMZ medium 503) [58]. All ingredients were dissolved in water except NH_4_Cl and KH_2_PO_4_, which were added after autoclaving from a sterile stock solution. The pH of the medium was adjusted to 7.5 – 7.7 prior to autoclaving. For incubation of cultures in closed serum vials under defined gas atmospheres the SYPHC medium was slightly modified: All compounds, except the HEPES buffer which was omitted, were dissolved in water and then the solution was sparged with a 80% N_2_ and 20% CO_2_ gas mixture for 45 min to remove dissolved oxygen. Various concentrations of oxygen in the headspace gas atmosphere were obtained by filling serum vials with anoxic medium to certain levels as described previously [8]. The pH of the medium was adjusted to 7.3 – 7.5 after autoclaving by adding Na_2_CO_3_ from a sterile and anoxic stock solution (5% w/v) that was prepared under a 80% N_2_ and 20% CO_2_ gas atmosphere. In some experiments the sodium pyruvate in SYPHC medium was replaced with sodium DL-malate and the resulting medium was designated SYMHC or SYM, if the amino acids L-histidine and L-cysteine were omitted.

All chemicals were obtained from Sigma-Aldrich (Taufkirchen, Germany) and complex nutrients from DIFCO BBL (Becton Dickinson; Heidelberg, Germany).

### Determination of growth and phenotypic traits

The absorbance values of growing cultures were determined in a Thermo Scientific BioMate 6 split beam UV/visible spectrophotometer using 1 cm light path disposable cuvettes and water as blank. The A_660nm_ reading was used to estimate the cell density. Expression of the light-harvesting complex in strain Ivo14^T^ was estimated by determining the A_870nm_ to A_660nm_ ratio, whereas for cultures of *C. litoralis* and *Chromatocurvus halotolerans* a ratio of A_880nm_ to A_660nm_ was used and for *H. rubra* a ratio of A_820nm_ to A_660nm_. The cellular dry weight of grown cultures was determined by overnight freeze-drying of cell pellets harvested by centrifugation. A comparison of the determined cellular dry weights with corresponding absorbance values revealed similar ratios for the strains Ivo14^T^, *Chromatocurvus halotolerans* DSM 23344^T^ and *H. rubra* DSM 19751^T^ grown in defined medium with pyruvate as carbon source (0.59, 0.59 and 0.58 mg dry weight per absorbance unit (A) at 660 nm, respectively). Significantly higher ratios were obtained upon cultivation of these strains in complex media containing malate and yeast extract, which may be due to the storage of reserve polymers. The corresponding values for strains Ivo14^T^, DSM 23344^T^ and DSM 19751^T^ were 0.68, 0.74 and 0.85 mg dry weight per A_660nm_.

The substrate utilization patterns of strains Ivo14^T^ and *H. rubra* DSM 19751^T^ were determined in SYPHC medium that was modified by omitting yeast extract and pyruvate. Without additional carbon source no growth took place in this medium. The defined medium described by Spring et al. [8] for testing carbon source utilization in *C. litoralis* was also used to test growth of *Chromatocurvus halotolerans* on single carbon sources. Carbon sources were added in various concentrations that depended on the approximate size of the respective molecule: 20 mM (1-2 carbon atoms), 10 mM (3-4 carbon atoms), 5 mM (5-6 carbon atoms), 2.5 mM (7-8 carbon atoms) and 1 mM (>9 carbon atoms). Growth on a carbon source was verified by measurements of the optical density in aliquots of the culture in intervals of two or three days until stationary phase was reached. At least one subsequent transfer in medium with the same carbon source was done to exclude a carryover of remaining substrates along with the inoculum in the first transfer. The growth response on a single carbon source was designated as negative, if the obtained OD_660_ value was below 0.05; as weak, if the maximal OD_660_ value was between 0.05 and 0.10; and positive, if it was above 0.10.

Sensitivity to antibiotics was determined by disk diffusion assays (Kirby-Bauer method) using the antimicrobial susceptibility disks offered by Oxoid (Wesel, Germany). The following antibiotics and concentrations were used: cephalotin (30 μg), imipenem (10 μg), chloramphenicol (10 μg), gentamicin (10 μg), neomycin (30 μg), colistin (10 μg), polymyxin B (300 units), oxacillin (5 μg), tetracycline (30 μg), doxycycline (30 μg), vancomycin (30 μg), lincomycin (15 μg), and bacitracin (10 units).

Characterization of additional morphological traits and diagnostic tests for enzymes and physiological activities were carried out as described previously [8]. Carbohydrates as reserve compound were detected in wet cell pellets by reaction with the anthrone reagent as reported elsewhere [59]. Tests were performed in duplicate including respective positive and negative controls. Unless noted otherwise all physiological tests were incubated at 28°C in dim light and at 12% (v/v) oxygen in the head space gas atmosphere.

### Analyses of pigments and cytochromes

Photosynthetic pigments were extracted from wet cell pellets using a mixture of acetone/methanol (7:2) as described previously [8]. Spectra were recorded with a Thermo Scientific BioMate 6 split beam UV/visible spectrophotometer. The concentrations of bacteriopheophytin *a*, bacteriochlorophyll *a* and spirilloxanthin in the acetone/methanol extracts were determined from the absorbance values obtained at 747, 771 and 475 nm, respectively, using the spectral reconstruction method of van der Rest and Gingras [60].

The detection and identification of various cytochrome types was done as reported previously [8].

### Chemotaxonomical characterization

Cellular fatty acid patterns were determined from cells grown to stationary phase in SYPHC liquid medium or on Marine Agar 2216. The preparation and extraction of fatty acid methyl esters from biomass and their subsequent separation and identification by gas chromatography was done as described elsewhere [61]. Respiratory lipoquinone and polar lipid analyses were carried out by the Identification Service and Dr. B.J. Tindall, DSMZ, Braunschweig, Germany, according to the protocols given by the DSMZ Identification Service [62].

### Detection of specific genes using PCR

For the isolation of genomic DNA from strain Ivo14^T^ and further reference strains the MasterPure™ Gram Positive DNA Purification Kit from Epicentre (Madison, USA) was used according to the instructions provided by the manufacturer. Extracted genomic DNA was quantified using a NanoDrop ND1000 spectrophotometer (Peqlab; Erlangen, Germany).

PCR amplification of genomic DNA was carried out using the HotMasterMix 2.5x from 5 PRIME (Hamburg, Germany) according to the manufacturer’s protocol or the *Taq* DNA polymerase from Qiagen (Hilden, Germany) in reaction buffer containing 200 μM (each) deoxynucleotide triphosphates (dNTPs), 1 μM (each) oligonucleotide primers and ca. 10 – 25 ng of genomic DNA in a final volume of 20 μl. PCR products were purified using the HiYield Gel/PCR clean-up and Gel-Extraction Kit (SLG; Gauting, Germany) according to the manufacturer’s protocol and visualized by gel electrophoresis (1% agarose). Finally, PCR products were sequenced using a BigDye Terminator v3.1 Cycle Sequencing kit (Life Technologies; Darmstadt, Germany) and an ABI 3730*xl* DNA Analyzer (Applied Biosystems; Darmstadt, Germany).

#### Amplification of pufLM genes

For detection of *pufL* and *pufM* genes in extracted DNA a PCR amplification was performed with two sets of degenerated primers (see Table 4). Sequences of the primer set pufLF2/pufMR2 were optimized to match known sequences of BChl *a*-containing members of the OM60/NOR5 clade. The amplification comprises the following program: an initial step at 98°C for 3 min and then 35 cycles at 98°C for 15 s, 56°C for 25 s and 72°C for 1.5 min. At the end a postelongation at 72°C for 10 min was carried out.

**Table 4:** Oligonucleotides used for the amplification of gene fragments with PCR

**Primer**	** Sequence (5′-3′)**	**T**_**a **_**(°C)**	**Protein encoded by the target gene**	**Product size (bp)**	**Reference**
**pufLF1**	CTK TTC GAC TCC TGG GTS GG	56	Reaction center proteins L and M subunits	1500	[5]
**pufMR1**	CCA TSG TCC AGC GCC AGA A				
**pufLF2**	CTY TTT GAY TTC TGG GTD GG	56	Reaction center proteins L and M subunits	1500	This study
**pufMR2**	CCA TSG TCC AGC GCC ARA A				
**PR1**	MGN TAY ATH GAY TGG YT	47	Proteorhodopsin opsin subunit	312	[26]
**PR2**	WWN MGN TAY GTN GAY TGG				
**PR3**	GGR TAD ATN GCC CAN CC				
**soxB432F-2**	GAY GGN GGN GAY MYB TGG	54	Sox enzyme complex B subunit	1000	This study
**soxB1446B-2**	CAT RTC WCC MCC YTG YTG				
**rpoB-F**	AAY CAG TTC CGC GTN GGH YTN GT	52	RNA polymerase beta subunit	1000	This study
**rpoB-R**	AAG TTR TAR CCR TTC CAR GGC AT				

The IUPAC nucleotide code is used to indicate wobble positions in degenerate primer sequences. T_a_ indicates the annealing temperature used in the PCR reaction.

#### Amplification of proteorhodopsin genes

For detection of proteorhodopsin genes in genomic DNA samples the degenerate primers PR1, PR2, and PR3 (see Table 4) targeted against most known proteorhodopsin genes were used to perform a multiplex PCR analysis. The amplification comprises the following program: an initial step at 94°C for 1 min and then 35 cycles at 94°C for 10 s, 47°C for 30 s and 68°C for 50 s. At the end a postelongation at 68°C for 1.5 min was carried out.

#### Amplification of soxB genes

For detection of the sulfate thiol esterase subunit (SoxB) of the periplasmic sox enzyme complex the primers soxB432F-2 and soxB1446B-2 were designed, which are based on primers proposed previously [63], but with some modifications to match known *soxB* gene sequences of representatives belonging to the OM60/NOR5 clade. For amplification the protocol was carried out as described for the *pufLM* primer except that an annealing temperature of 54°C was used.

#### Amplification of rpoB genes

Primers used for the amplification of *rpoB* fragments with an expected size of around 1000 nucleotides were designed based on an alignment of complete *rpoB* sequences of strains belonging to the OM60/NOR5 clade (Table 4). For amplification the protocol was carried out as described for the *pufLM* primers except that an annealing temperature of 52°C was used.

### Genome sequencing and phylogenetic analyses

As part of the Moore Foundation Microbial Genome Sequencing Project [64] the genomes of Rap1red and Ivo14^T^ were shotgun sequenced by the J. Craig Venter Institute (JCVI). Two genomic libraries with insert sizes of 1 - 4 kb and 10 - 12 kb were made and sequenced from both ends to provide paired-end reads on ABI 3730*xl* DNA sequencers (Applied Biosystems, Foster City, CA) to approx. 8× coverage. The draft genomes of Rap1red (= NOR5-3) and Ivo14^T^ (= NOR5-1B^T^) are deposited under GenBank accession numbers ACCX01000000 and ACCY01000000, respectively. A genome report compliant with the “Minimum Information about a Genome Sequence specification” is available from the Genomes Online Database [65]. The genome sequences were all automatically annotated by JCVI. These sequences were imported into the GenDB gene annotation system [66,67] and the genes were further analyzed. Despite the automatic annotations, all the gene findings in this study were based on manual gene comparison rather than automatic annotation, since in several cases the automated annotation was incorrect. In order to determine whether a gene has homologs existing in other genomes, we used the *genomic* BLAST tool of the NCBI [68] with the tblastn (search translated nucleotide database using a protein query) algorithm for searching.

The Genome-To-Genome Distance Calculator [69] was used for genome-based species delineation as described [70]. This system calculates DNA-DNA similarity values by comparing the genomes to obtain high-scoring segment pairs (HSPs) and inferring distances from a set of three formulas (1, HSP length/total length; 2, identities/HSP length; 3, identities/total length). Spectroscopic DNA-DNA reassociation experiments were performed according to the protocol outlined by the DSMZ Identification Service [62].

Phylogenetic trees based on 16S rRNA, *pufLM* and *rpoB* gene sequences were reconstructed using distance matrix (neighbor-joining) and parsimony programs included in the ARB package [71]. Maximum likelihood trees were reconstructed with the program RAxML (version 7.2.8) using raxmlGUI [72] and the GTRGAMMA option with 1000 rounds of bootstrap replicates [73]. The dataset of aligned and almost complete 16S rRNA gene sequences was based on the ARB SILVA database release 108 (September 2011) [74], whereas DNA sequences of *pufL*, *pufM* and *rpoB* genes were obtained from GenBank and aligned using the ClustalW algorithm implemented in the ARB package. The generated alignments of *pufLM* and *rpoB* nucleotide sequences in PHYLIP format are available as Additional file 2 and Additional file 3, respectively. Identity values of aligned nucleotide sequences were determined by using the similarity option of the neighbor-joining program included in the ARB package.

## Competing interests

The authors declare that they have no competing interests.

## Authors’ contributions

BMF and SS developed the study concept. SS conceived and designed a majority of the experiments. SS and TR performed the experiments. BMF, SY, JH, TR and CS contributed materials and analysis tools. SS wrote the paper. All authors read and approved the final manuscript.

## Supplementary Material

Additional file 1: Table S1 Cellular fatty acid patterns of strain Ivo14^T^, *Chromatocurvus halotolerans* DSM 23344^T^, *Pseudohaliea* (= *Haliea*) *rubra* DSM 19751^T^ and *Congregibacter litoralis* DSM 17192^T^ in correlation to the oxygen concentration in the head space gas atmosphere during growth in SYPHC medium. The fatty acid nomenclature is explained in the legend of Table 2 in the main text. The abundance of unsaturated fatty acids that may depend on the activity of desaturases for their synthesis are given in red color. Click here for file

Additional file 2 **Alignment of *pufL* and *pufM* nucleotide sequences in PHYLIP format used to reconstruct the phylogenetic dendrogram shown in Figure **3**A.** Click here for file

Additional file 3 **Alignment of *rpoB* nucleotide sequences in PHYLIP format used to reconstruct the phylogenetic dendrogram shown in Figure **3**B.** Click here for file

## References

[B1] Kolber ZS Plumley FG Lang AS Beatty JT Blankenship RE VanDover CL Vetriani C Koblížek M Rathenberg C Falkowski PG Contribution of aerobic photoheterotrophic bacteria to the carbon cycle in the Ocean Science 2001 292 2492 2495 10.1126/science.1059707 11431568

[B2] Yutin N Suzuki MT Teeling H Weber M Venter JC Rusch DB Béjà O Assessing diversity and biogeography of aerobic anoxygenic phototrophic bacteria in surface waters of the Atlantic and Pacific Oceans using the Global Ocean Sampling expedition metagenomes Environ Microbiol 2007 9 1464 1475 10.1111/j.1462-2920.2007.01265.x 17504484

[B3] Wagner-Döbler I Biebl H Environmental biology of the marine *Roseobacter* lineage Ann Rev Microbiol 2006 60 255 280 10.1146/annurev.micro.60.080805.142115 16719716

[B4] Yurkov V Dworkin M, Falkow S, Rosenberg E, Schleifer K-H, Stackebrandt E Aerobic phototrophic proteobacteria The Prokaryotes. Volume 5 2006 3 New York: Springer 562 584

[B5] Béjà O Suzuki MT Heidelberg JF Nelson WC Preston CM Hamada T Eisen JA Fraser CM DeLong EF Unsuspected diversity among marine aerobic anoxygenic phototrophs Nature 2002 415 630 633 10.1038/415630a 11832943

[B6] Cho J-C Stapels MD Morris RM Vergin KL Schwalbach MS Givan SA Barofsky DF Giovannoni SJ Polyphyletic photosynthetic reaction centre genes in oligotrophic marine *Gammaproteobacteria* Environ Microbiol 2007 9 1456 1463 10.1111/j.1462-2920.2007.01264.x 17504483

[B7] Fuchs BM Spring S Teeling H Quast C Wulf J Schattenhofer M Yan S Ferriera S Johnson J Glöckner FO Amann R Characterization of a marine gammaproteobacterium capable of aerobic anoxygenic photosynthesis Proc Natl Sci USA 2007 104 2891 2896 10.1073/pnas.0608046104 PMC1815277 17299055

[B8] Spring S Lünsdorf H Fuchs BM Tindall BJ The photosynthetic apparatus and its regulation in the aerobic gammaproteobacterium *Congregibacter litoralis* gen. nov., sp. nov PLoS One 2009 4 3 e4866 10.1371/journal.pone.0004866 19287491 PMC2654016

[B9] Cho J-C Giovannoni SJ Cultivation and growth characteristics of a diverse group of oligotrophic marine *Gammaproteobacteria* Appl Environ Microbiol 2004 70 432 440 10.1128/AEM.70.1.432-440.2004 14711672 PMC321273

[B10] Rappé MS Kemp PF Giovannoni SJ Phylogenetic diversity of marine coastal picoplankton 16S rRNA genes cloned from the continental shelf off Cape Hatteras, North Carolina Limnol Oceanogr 1997 42 811 826 10.4319/lo.1997.42.5.0811

[B11] Eilers H Pernthaler J Peplies J Glöckner FO Gerdts G Amann R Isolation of novel pelagic bacteria from the German Bight and their seasonal contributions to surface picoplankton Appl Environ Microbiol 2001 67 5134 5142 10.1128/AEM.67.11.5134-5142.2001 11679337 PMC93282

[B12] Alonso-Sáez L Balagué V Sà EL Sánchez O González JM Pinhassi J Massana R Pernthaler J Pedrós-Alió C Gasol JM Seasonality in bacterial diversity in north-west Mediterranean coastal waters: assessment through clone libraries, fingerprinting and FISH FEMS Microbiol Ecol 2007 60 98 112 10.1111/j.1574-6941.2006.00276.x 17250750

[B13] Yan S Fuchs BM Lenk S Harder J Wulf J Jiao NZ Amann R Biogeography and phylogeny of the NOR5/OM60 clade of *Gammaproteobacteria* Syst Appl Microbiol 2009 32 124 139 10.1016/j.syapm.2008.12.001 19216045

[B14] Jiao N Zhang Y Zeng Y Hong N Liu R Chen F Wang P Distinct distribution pattern of abundance and diversity of aerobic anoxygenic phototrophic bacteria in the global ocean Environ Microbiol 2007 9 3091 3099 10.1111/j.1462-2920.2007.01419.x 17991036

[B15] Csotonyi JT Swiderski J Stackebrandt E Yurkov VV Novel halophilic aerobic anoxygenic phototrophs from a Canadian hypersaline spring system Extremophiles 2008 12 529 539 10.1007/s00792-008-0156-8 18385928

[B16] Jang Y Oh HM Kang I Lee K Yang SJ Cho JC Genome sequence of strain IMCC3088, a proteorhodopsin-containing marine bacterium belonging to the OM60/NOR5 clade J Bacteriol 2011 193 3415 3416 10.1128/JB.05111-11 21551310 PMC3133269

[B17] Lucena T Pascual J Garay E Arahal DR Macián MC Pujalte MJ *Haliea mediterranea* sp. nov., a marine gammaproteobacterium Int J Syst Evol Microbiol 2010 60 1844 1848 10.1099/ijs.0.017061-0 19767360

[B18] Urios L Intertaglia L Lesongeur F Lebaron P *Haliea rubra* sp. nov., a member of the *Gammaproteobacteria* from the Mediterranean Sea Int J Syst Evol Microbiol 2009 59 1188 1192 10.1099/ijs.0.002220-0 19406817

[B19] Urios L Intertaglia L Lesongeur F Lebaron P *Haliea salexigens* gen. nov., sp. nov., a member of the *Gammaproteobacteria* from the Mediterranean Sea Int J Syst Evol Microbiol 2008 58 1233 1237 10.1099/ijs.0.65470-0 18450719

[B20] Park S Yoshizawa S Inomata K Kogure K Yokota A *Halioglobus japonicus* gen. nov., sp. nov., and *Halioglobus pacificus* sp. nov., members of the class *Gammaproteobacteria* isolated from seawater Int J Syst Evol Microbiol 2012 62 1784 1789 10.1099/ijs.0.031443-0 21986723

[B21] Lee YK Hong SG Cho HH Cho KH Lee HK *Dasania marina* gen. nov., sp. nov., of the order *Pseudomonadales*, isolated from Arctic marine sediment J Microbiol 2007 45 505 509 18176532

[B22] Park S Yoshizawa S Kogure K Yokota A *Oceanicoccus sagamiensis* gen. nov., sp. nov., a gammaproteobacterium isolated from sea water of Sagami Bay in Japan J Microbiol 2011 49 233 237 10.1007/s12275-011-0368-y 21538243

[B23] Graeber I Kaesler I Borchert MS Dieckmann R Pape T Lurz R Nielsen P von Döhren H Michaelis W Szewzyk U *Spongiibacter marinus* gen. nov., sp. nov., a halophilic marine bacterium isolated from the boreal sponge *Haliclona* sp. 1 Int J Syst Evol Microbiol 2008 58 585 590 10.1099/ijs.0.65438-0 18319460

[B24] Li HJ Zhang XY Chen CX Zhang YJ Gao ZM Yu Y Chen XL Chen B Zhang YZ *Zhongshania antarctica* gen. nov., sp. nov. and *Zhongshania guokunii* sp. nov., gammaproteobacteria respectively isolated from coastal attached (fast) ice and surface seawater of the Antarctic Int J Syst Evol Microbiol 2011 61 2052 2057 10.1099/ijs.0.026153-0 20851909

[B25] Winkelmann N Harder J An improved isolation method for attached-living *Planctomycetes* of the genus *Rhodopirellula* J Microbiol Methods 2009 77 276 284 10.1016/j.mimet.2009.03.002 19303037

[B26] Sabehi G Loy A Jung KH Partha R Spudich JL Isaacson T Hirschberg J Wagner M Béjà O New insights into metabolic properties of marine bacteria encoding proteorhodopsins PLoS Biol 2005 3 e273 10.1371/journal.pbio.0030273 16008504 PMC1175822

[B27] Riedel T Tomasch J Buchholz I Jacobs J Kollenberg M Gerdts G Wichels A Brinkhoff T Cypionka H Wagner-Döbler I Constitutive expression of the proteorhodopsin gene by a flavobacterium strain representative of the proteorhodopsin-producing microbial community in the North Sea Appl Environ Microbiol 2010 76 3187 3197 10.1128/AEM.02971-09 20305030 PMC2869143

[B28] Steindler L Schwalbach MS Smith DP Chan F Giovannoni SJ Energy starved *Candidatus* Pelagibacter ubique substitutes light-mediated ATP production for endogenous carbon respiration PLoS One 2011 6 e19725 10.1371/journal.pone.0019725 21573025 PMC3090418

[B29] Cogdell RJ Durant I Valentine J Lindsay JG Schmidt K The isolation and partial characterisation of the light-harvesting pigment-protein complement of *Rhodopseudomonas acidophila* Biochim Biophys Acta 1983 722 427 435 10.1016/0005-2728(83)90058-0

[B30] McLuskey K Prince SM Cogdell RJ Isaacs NW The crystallographic structure of the B800-820 LH3 light-harvesting complex from the purple bacteria *Rhodopseudomonas acidophila* Strain 7050 Biochemistry 2001 40 8783 8789 10.1021/bi010309a 11467938

[B31] Csotonyi JT Stackebrandt E Swiderski J Schumann P Yurkov V *Chromocurvus halotolerans* gen. nov., sp. nov., a gammaproteobacterial obligately aerobic anoxygenic phototroph, isolated from a Canadian hypersaline spring Arch Microbiol 2011 193 573 582 10.1007/s00203-011-0698-5 21479531

[B32] Spring S Riedel T Mixotrophic growth of bacteriochlorophyll *a*-containing members of the OM60/NOR5 clade of marine gammaproteobacteria is carbon-starvation independent and correlates with the cellular redox state BMC Microbiol 2013 13 117 10.1186/1471-2180-13-117 23705861 PMC3666943

[B33] Bonomo J Gill RT Amino acid content of recombinant proteins influences the metabolic burden response Biotechnol Bioeng 2005 90 116 126 10.1002/bit.20436 15736162

[B34] Shand RF Blum PH Mueller RD Riggs DL Artz SW Correlation between histidine operon expression and guanosine 5′-diphosphate-3′-diphosphate levels during amino acid downshift in stringent and relaxed strains of *Salmonella typhimurium* J Bacteriol 1989 171 737 743 2492514 10.1128/jb.171.2.737-743.1989 PMC209659

[B35] Zhang YM Rock CO Membrane lipid homeostasis in bacteria Nat Rev Microbiol 2008 6 222 233 10.1038/nrmicro1839 18264115

[B36] Tomasch J Gohl R Bunk B Diez MS Wagner-Döbler I Transcriptional response of the photoheterotrophic marine bacterium *Dinoroseobacter shibae* to changing light regimes ISME J 2011 5 1957 1968 10.1038/ismej.2011.68 21654848 PMC3223308

[B37] Tank M Thiel V Imhoff JF Phylogenetic relationship of phototrophic purple sulfur bacteria according to *pufL* and *pufM* genes Int Microbiol 2009 12 175 185 19784924

[B38] Petersen J Brinkmann H Bunk B Michael V Päuker O Pradella S Think pink: photosynthesis, plasmids and the *Roseobacter* clade Environ Microbiol 2012 14 2661 2672 10.1111/j.1462-2920.2012.02806.x 22732061

[B39] Thrash JC Cho JC Ferriera S Johnson J Vergin KL Giovannoni SJ Genome sequences of strains HTCC2148 and HTCC2080, belonging to the OM60/NOR5 clade of the *Gammaproteobacteria* J Bacteriol 2010 192 3842 3843 10.1128/JB.00511-10 20472793 PMC2897341

[B40] Dufresne A Garczarek L Partensky F Accelerated evolution associated with genome reduction in a free-living prokaryote Genome Biol 2005 6 2 R14 10.1186/gb-2005-6-2-r14 15693943 PMC551534

[B41] Giovannoni SJ Tripp HJ Givan S Podar M Vergin KL Baptista D Bibbs L Eads J Richardson TH Noordewier M Rappé MS Short JM Carrington JC Mathur EJ Genome streamlining in a cosmopolitan oceanic bacterium Science 2005 309 1242 1245 10.1126/science.1114057 16109880

[B42] Maeda T Hayakawa K You M Sasaki M Yamaji Y Furushita M Shiba T Characteristics of nonylphenol polyethoxylate-degrading bacteria isolated from coastal sediments Microbes Environ 2005 20 253 257 10.1264/jsme2.20.253

[B43] Giovannoni SJ Bibbs L Cho JC Stapels MD Desiderio R Vergin KL Rappé MS Laney S Wilhelm LJ Tripp HJ Mathur EJ Barofsky DF Proteorhodopsin in the ubiquitous marine bacterium SAR11 Nature 2005 438 82 85 10.1038/nature04032 16267553

[B44] Stingl U Desiderio RA Cho JC Vergin KL Giovannoni SJ The SAR92 clade: an abundant coastal clade of culturable marine bacteria possessing proteorhodopsin Appl Environ Microbiol 2007 73 2290 2296 10.1128/AEM.02559-06 17293499 PMC1855678

[B45] Gómez-Consarnau L Akram N Lindell K Pedersen A Neutze R Milton DL González JM Pinhassi J Proteorhodopsin phototrophy promotes survival of marine bacteria during starvation PLoS Biol 2010 8 e1000358 10.1371/journal.pbio.1000358 20436956 PMC2860489

[B46] Morris RM Rappé MS Connon SA Vergin KL Siebold WA Carlson CA Giovannoni SJ SAR11 clade dominates ocean surface bacterioplankton communities Nature 2002 420 806 810 10.1038/nature01240 12490947

[B47] Ritchie AE Johnson ZI Abundance and genetic diversity of aerobic anoxygenic phototrophic bacteria of coastal regions of the Pacific Ocean Appl Environ Microbiol 2012 78 2858 2866 10.1128/AEM.06268-11 22307290 PMC3318826

[B48] Schwalbach MS Fuhrmann JA Wide-ranging abundances of aerobic anoxygenic phototrophic bacteria in the world ocean revealed by epifluorescence microscopy and quantitative PCR Limnol Oceanogr 2005 50 620 628 10.4319/lo.2005.50.2.0620

[B49] Stackebrandt E Ebers J Taxonomic parameters revisited: tarnished gold standards Microbiol Today 2006 33 152 155

[B50] Stackebrandt E Goebel BM A place for DNA–DNA reassociation and 16S rRNA sequence analysis in the present species definition in bacteriology Int J Syst Bacteriol 1994 44 846 849 10.1099/00207713-44-4-846

[B51] Pitcher DG Windsor D Windsor H Bradbury JM Yavari C Jensen JS Ling C Webster D *Mycoplasma amphoriforme* sp. nov., isolated from a patient with chronic bronchopneumonia Int J Syst Evol Microbiol 2005 55 2589 2594 10.1099/ijs.0.63269-0 16280532

[B52] Pikuta EV Hoover RB Bej AK Marsic D Whitman WB Krader P *Spirochaeta dissipatitropha* sp. nov., an alkaliphilic, obligately anaerobic bacterium, and emended description of the genus *Spirochaeta* Ehrenberg 1835 Int J Syst Evol Microbiol 2009 59 1798 1804 19578151 10.1099/ijs.0.65862-0

[B53] Anil Kumar P Srinivas TN Thiel V Tank M Sasikala C Ramana CV Imhoff JF *Thiohalocapsa marina* sp. nov., from an Indian marine aquaculture pond Int J Syst Evol Microbiol 2009 59 2333 2338 10.1099/ijs.0.003053-0 19620368

[B54] Giammanco GM Grimont PA Grimont F Lefevre M Giammanco G Pignato S Phylogenetic analysis of the genera *Proteus*, *Morganella* and *Providencia* by comparison of *rpoB* gene sequences of type and clinical strains suggests the reclassification of *Proteus myxofaciens* in a new genus, *Cosenzaea* gen. nov., as *Cosenzaea myxofaciens* comb. nov Int J Syst Evol Microbiol 2011 61 1638 1644 10.1099/ijs.0.021964-0 20709916

[B55] Adékambi T Drancourt M Raoult D The *rpoB* gene as a tool for clinical microbiologists Trends Microbiol 2009 17 37 45 10.1016/j.tim.2008.09.008 19081723

[B56] Adékambi T Shinnick TM Raoult D Drancourt M Complete *rpoB* gene sequencing as a suitable supplement to DNA–DNA hybridization for bacterial species and genus delineation Int J Syst Evol Microbiol 2008 58 1807 1814 10.1099/ijs.0.65440-0 18676461

[B57] Euzéby J Validation list no. 145: List of new names and new combinations previously effectively, but not validly, published Int J Syst Evol Microbiol 2012 62 1017 1019 10.1099/ijs.0.64643-0 16957093

[B58] DSMZ Catalogue Microorganisms http://www.dsmz.de/catalogues/catalogue-microorganisms/culture-technology.html] (accessed May 15, 2013)

[B59] Brooks KK Liang B Watts JL The Influence of bacterial diet on fat storage in *C. elegans* PLoS ONE 2009 4 10 e7545 10.1371/journal.pone.0007545 19844570 PMC2760100

[B60] Van der Rest M Gingras G The pigment complement of the photosynthetic reaction center isolated from *Rhodospirillum rubrum* J Biol Chem 1974 249 6446 6453 4214257

[B61] Kaksonen AH Spring S Schumann P Kroppenstedt RM Puhakka JA *Desulfotomaculum thermosubterraneum* sp. nov., a thermophilic sulfate-reducer isolated from an underground mine located in geothermally active area Int J Syst Evol Microbiol 2006 56 2603 2608 10.1099/ijs.0.64439-0 17082399

[B62] Identification and characterization of microorganisms and cultures http://www.dsmz.de/services/services-microorganisms/identification.html] (accessed May 15, 2013)

[B63] Petri R Podgorsek L Imhoff JF Phylogeny and distribution of the *soxB* gene among thiosulfate-oxidizing bacteria FEMS Microbiol Lett 2001 197 171 178 10.1111/j.1574-6968.2001.tb10600.x 11313131

[B64] Moore Foundation Microbial Genome Sequencing Project http://camera.calit2.net/microgenome/] (accessed May 15, 2013)

[B65] Genomes Online Database http://www.genomesonline.org] (accessed May 15, 2013)

[B66] GenDB gene annotation system http://www2.cebitec.uni-bielefeld.de/comics/index.php/gendb/] (accessed May 15, 2013)

[B67] Meyer F Goesmann A McHardy AC Bartels D Bekel T Clausen J Kalinowski J Linke B Rupp O Giegerich R Pühler A GenDB-an open source genome annotation system for prokaryote genomes Nucleic Acids Res 2003 31 2187 2195 10.1093/nar/gkg312 12682369 PMC153740

[B68] NCBI BLAST tool http://www.ncbi.nlm.nih.gov/sutils/genom_table.cgi] (accessed May 15, 2013)

[B69] GGDC - Genome-To-Genome Distance Calculator http://ggdc.gbdp.org/] (accessed May 15, 2013)

[B70] Auch AF von Jan M Klenk HP Göker M Digital DNA-DNA hybridization for microbial species delineation by means of genome-to-genome sequence comparison Stand Genomic Sci 2010 2 117 134 10.4056/sigs.531120 21304684 PMC3035253

[B71] Ludwig W Strunk O Westram R Richter L Meier H Yadhukumar Buchner A Lai T Steppi S Jobb G Förster W Brettske I Gerber S Ginhart AW Gross O Grumann S Hermann S Jost R König A Liss T Lüssmann R May M Nonhoff B Reichel B Strehlow R Stamatakis A Stuckmann N Vilbig A Lenke M Ludwig T Bode A Schleifer KH ARB: a software environment for sequence data Nucleic Acids Res 2004 32 1363 1371 10.1093/nar/gkh293 14985472 PMC390282

[B72] Silvestro D Michalak I raxmlGUI: a graphical front-end for RAxML Org Divers Evol 2012 12 335 337 10.1007/s13127-011-0056-0

[B73] Stamatakis A RAxML-VI-HPC: maximum likelihood-based phylogenetic analyses with thousands of taxa and mixed models Bioinformatics 2006 22 2688 2690 10.1093/bioinformatics/btl446 16928733

[B74] Pruesse E Quast C Knittel K Fuchs B Ludwig W Peplies J Glöckner F SILVA: a comprehensive online resource for quality checked and aligned ribosomal RNA sequence data compatible with ARB Nucleic Acids Res 2007 35 7188 7196 10.1093/nar/gkm864 17947321 PMC2175337

