# Supplementary material for: Taxonomy and evolution of bacteriochlorophyll a-containing members of the OM60/NOR5 clade of marine gammaproteobacteria: description of Luminiphilus syltensis gen. nov., sp. nov., reclassification of Haliea rubra as Pseudohaliea rubra gen. nov., comb. nov., and emendation of Chromatocurvus halotolerans
Source: BMC Microbiol. 2013 May 24;13:118. doi: 10.1186/1471-2180-13-118 (PMC3679898; doi:10.1186/1471-2180-13-118)
Supplement: Additional file 1: Table S1 — Cellular fatty acid patterns of strain Ivo14T, Chromatocurvus halotolerans DSM 23344T, Pseudohaliea (= Haliea) rubra DSM 19751T and Congregibacter litoralis DSM 17192T in correlation to the oxygen concentration in the head space gas atmosphere during growth in SYPHC medium. The fatty acid nomenclature is explained in the legend of Table 2 in the main text. The abundance of unsaturated fatty acids that may depend on the activity of desaturases for their synthesis are given in red color. [file 1471-2180-13-118-S1.doc]

**Supplementary Table. Cellular fatty acid patterns of *Luminiphilus syltensis* Ivo14^T^, *Chromatocurvus halotolerans* DSM 23344^T^, *Pseudohaliea* (= *Haliea*) *rubra* DSM 19751^T^ and *Congregibacter litoralis* DSM 17192^T^ in correlation to the oxygen concentration in the head space gas atmosphere during growth in SYPHC medium.** The fatty acid nomenclature is explained in the legend of Table 2 in the main text. The abundance of unsaturated fatty acids that may depend on the activity of desaturases for their synthesis are given in red color.

| **Fatty acid** | **Ivo14^T^** | | |  | **DSM 23344^T^** | | |  | **DSM 19751^T^** | | |  | **DSM 17192^T^** | | |
| --- | --- | --- | --- | --- | --- | --- | --- | --- | --- | --- | --- | --- | --- | --- | --- |
|  | **Air** | **O_2_< 12%** | **O_2_< 6%** |  | **Air** | **O_2_< 12%** | **O_2_< 6%** |  | **Air** | **O_2_< 12%** | **O_2_< 6%** |  | **Air** | **O_2_< 12%** | **O_2_< 6%** |
| ***Saturated fatty acids*** |  |  |  |  |  |  |  |  |  |  |  |  |  |  |  |
| 10:0 | - | - | - |  | - | 0.6 | - |  | - | - | - |  | - | - | - |
| 12:0 | **5.2** | 2.5 | 3.4 |  | 0.8 | 2.0 | 2.0 |  | - | - | - |  | 3.0 | 2.1 | 2.1 |
| 13:0 | - | - | - |  | - | - | - |  | - | - | - |  | - | - | - |
| 14:0 | 1.9 | 3.5 | 4.7 |  | 0.9 | 2.9 | 2.9 |  | - | - | 1.0 |  | 3.1 | 2.5 | 2.6 |
| 15:0 ISO | - | - | - |  | - | - | - |  | 0.6 | - | - |  | - | - | - |
| 15:0 | - | - | - |  | 0.8 | 2.5 | 3.2 |  | 0.6 | - | 1.4 |  | 1.0 | - | - |
| 16:0 | **33.0** | **33.2** | **35.9** |  | **17.7** | **22.7** | **24.3** |  | **11.5** | **11.0** | **12.5** |  | **11.6** | **12.7** | **12.0** |
| 17:0 ANTEISO | - | - | - |  | - | - | - |  | 1.3 | - | - |  | - | - | - |
| 17:0 | - | - | - |  | 1.0 | 1.8 | 2.1 |  | 1.0 | - | 0.7 |  | 1.3 | - | 0.7 |
| 17:0 10 methyl | - | - | - |  | - | - | - |  | - | - | 1.5 |  | - | 0.7 | 0.9 |
| 18:0 | 1.7 | 1.1 | 1.1 |  | 0.7 | 0.7 | 0.6 |  | 0.9 | 0.6 | 0.6 |  | 1.4 | 1.9 | 1.2 |
| ***Unsaturated fatty acids*** |  |  |  |  |  |  |  |  |  |  |  |  |  |  |  |
| 16:1 ω6c | - | - | - |  | - | - | - |  | - | - | - |  | **9.0** | **22.8** | **26.2** |
| 16:1 ω7c | **25.2** | **27.0** | **25.7** |  | **30.2** | **29.9** | **29.0** |  | **25.0** | **27.0** | **33.6** |  | **25.5** | **12.3** | **10.7** |
| 16:1 ω9c | - | - | - |  | - | - | - |  | 0.9 | 1.3 | - |  | - | - | - |
| 17:1 ω6c | - | - | - |  | - | - | - |  | 0.9 | 0.8 | 0.8 |  | - | - | - |
| 17:1 ω8c | - | - | - |  | 2.4 | 2.1 | 2.0 |  | 1.6 | 0.9 | 0.8 |  | 1.2 | - | - |
| 18:1 ω6c | - | - | - |  | - | - | - |  | - | - | - |  | - | **18.6** | **20.6** |
| 18:1 ω7c | **19.6** | **25.2** | **20.4** |  | **36.6** | **26.7** | **25.8** |  | **38.4** | **39.1** | **36.5** |  | **37.0** | **21.5** | **18.0** |
| 18:1 ω9c | - | - | - |  | - | - | - |  | **5.4** | **7.5** | - |  | - | - | - |
| 11 methyl 18:1 ω7c | - | 2.9 | 4.2 |  | - | 0.7 | 0.9 |  | - | - | - |  | - | 1.0 | 1.0 |
| ***Hydroxy fatty acids*** |  |  |  |  |  |  |  |  |  |  |  |  |  |  |  |
| 10:0 3OH | 4.8 | 1.9 | 2.6 |  | 0.8 | 1.0 | 1.0 |  | - | - | - |  | 2.6 | 1.7 | 1.6 |
| 12:0 2OH | - | - | - |  | 1.0 | - | - |  | 0.7 | 0.7 | 0.7 |  | - | - | - |
| 12:0 3OH | 2.4 | 1.1 | 1.3 |  | 0.9 | 1.0 | 1.0 |  | - | - | - |  | 1.1 | 0.9 | 0.8 |
| 12:1 3OH | - | - | - |  | 1.9 | 1.1 | 1.0 |  | 2.9 | 2.8 | 2.6 |  | - | - | - |
| **Sum in Feature 7** | 4.7 | 1.1 | - |  | 0.7 | - | - |  | 1.2 | - | - |  | 0.6 | - | - |
